# Parameter identifiability of linear-compartmental mammillary models

**DOI:** 10.1007/s11538-025-01568-1

**Published:** 2025-12-24

**Authors:** Katherine Clemens, Jonathan Martinez, Anne Shiu, Michaela Thompson, Benjamin Warren

**Affiliations:** 1 Bryn Mawr College, Bryn Mawr, PA USA; 2 University of Southern California, Los Angeles, CA USA; 3 Texas A&M University, College Station, TX USA; 4 Fairfield University, Fairfield, CT USA; 5 Present Address: Rochester Institute of Technology, Rochester, NY USA

**Keywords:** Linear compartmental model, Structural identifiability, Mammillary model, Bidirected-tree model, Input-output equation, Elementary symmetric polynomial, 93B30, 92C45, 37N25, 34A30, 34A55

## Abstract

Linear compartmental models are a widely used tool for analyzing systems arising in biology, medicine, and more. In such settings, it is essential to know whether model parameters can be recovered from experimental data. This is the identifiability problem. For a class of linear compartmental models with one input and one output, namely, those for which the underlying graph is a bidirected tree, Bortner *et al.* completely characterized which such models are structurally identifiability, which means that every parameter is generically locally identifiable. Here, we delve deeper, by examining which individual parameters are locally versus globally identifiable. Specifically, we analyze mammillary models, which consist of one central compartment which is connected to all other (peripheral) compartments. For these models, which fall into five infinite families, we determine which individual parameters are locally versus globally identifiable, and we give formulas for some of the globally identifiable parameters in terms of the coefficients of input-output equations. Our proofs rely on a combinatorial formula due to Bortner *et al.* for these coefficients.

## Introduction

Compartmental models are used frequently in applications ranging from ecology (Mulholland and Keener 1974) to disease modeling (Tang et al. 2020) and pharmacology (Cherruault and Sarin 1992). A well-known example arises in pharmacokinetics, in which linear compartmental models are widely used to predict the concentration of an injected drug in the body over time; such knowledge is essential for determining optimal drug dosage and timing. Linear compartmental models additionally have been used to describe the distribution of potassium between plasma and red blood cells, the distribution and turnover of cholesterol in the human body, and the flow of energy within ecosystems (Jacquez 1972).

In such applications, it is crucial to know whether the parameters of models can be uniquely determined from data (Massonis et al. 2021; Muñoz-Tamayo et al. 2018; Stigter 2024; Wieland et al. 2021). This is the problem of parameter *identifiability*. Our focus here is on *structural identifiability*, which assumes the ideal situation of noiseless data. Access to noiseless data is generally not realistic, but assessing structural identifiability is nevertheless of great importance, as it is a prerequisite for *practical identifiability*, which allows for data with noise.

The inspiration for our work is a recent classification of (generic) local identifiability for a family of linear compartmental models, namely, those in which the underlying graph is a bidirected tree. This classification is due to Bortner et al. (2023), and it states the following: *A bidirected-tree model with one input and one output is generically locally identifiable if and only if the model has at most 1 leak, and the distance from the input to output is at most 1*. In other words, this result tells us exactly which models have all parameters (at least) generically locally identifiable. Such models are called *identifiable*.

Bortner *et al.*’s result on bidirected-tree models motivates the following questions:

(**Q1**): In identifiable bidirected-tree models, which parameters are generically globally identifiable (that is, uniquely identifiable)?
(**Q2**): In unidentifiable bidirected-tree models, what is the source of this unidentifiability, that is, which parameters are unidentifiable?

The significance of question (**Q1**) in applications comes from the fact that being able to estimate parameters uniquely is preferable to being able to do so only up to a finite set. Similarly, regarding question (**Q2**), being able to estimate some but not all parameters may suffice in some applications, so we wish to know which ones can or can not be recovered.

Our work addresses questions (**Q1**) and (**Q2**) for an infinite family of bidirected-tree models. These models are *mammillary models*, which means that the underlying graph is a star graph, consisting of a central compartment connected to a number of peripheral compartments. Mammillary models are of “considerable practical importance” (Godfrey 1983, pg. 31), and instances of mammillary models in biological applications appear in Cherruault and Sarin (1992); Godfrey (1983); Jacquez (1972); and Matis et al. (1976); Nakashima and Benet (1989).

**Fig. 1:**
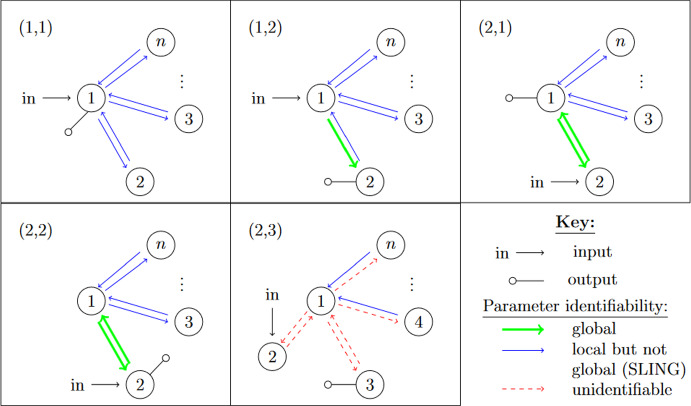
**Summary of main results**. All mammillary models (up to symmetry) with one input, one output, and no leaks are depicted. The model with input in compartment-*i* and output in compartment-*j* is labeled by (*i*, *j*). Identifiability properties of all parameters are shown; generically globally identifiable parameters are indicated by (green) boldface arrows, generically locally (but not globally) identifiable parameters are shown in (blue) plain arrows, and unidentifiable parameters are shown with (red) dashed arrows. These properties are proven or conjectured in this work (see Theorem 3.2 and Conjecture 3.18). The result for the model (1,1) was already known (Cobelli et al. 1979, §4.1)

Five (families of) mammillary models are depicted in Figure 1, which summarizes our main results. These results concern all mammillary models with one input, one output, and no leaks. Specifically, we prove the identifiability properties – generically globally identifiable versus generically locally identifiable versus unidentifiable – of most of the edge parameters, as shown in Figure 1 (Theorem 3.2). There are, however, several edges whose identifiability properties we verified computationally for several values of *n* (here, *n* denotes the number of compartments), but currently can not prove for all *n*. These edges are the edges in model (2, 3) that are marked as unidentifiable (indicated by dashed arrows) in Figure 1 (Conjecture 3.18).

In summary, for the models in Figure 1, we completely answer question (**Q1**) and we conjecture an answer to question (**Q2**). We emphasize that, to our knowledge, we are essentially the first to investigate questions (**Q1**) and (**Q2**) for infinite families of models. (Chau investigated similar questions for reparametrized versions of mammillary models with leaks at all compartments (Chau 1985b) and so-called catenary models (Chau 1985a); while Cobelli, Lepschy, and Romanin Jacur investigated the mammillary models we label by (1,1) and other models (Cobelli et al. 1979) – see also (Cobelli et al. 2002, §5.8) – but their focus was slightly different.) On the other hand, for models arising in biological applications (cellular signaling, epidemiology, physiology, and other areas), Barreiro and Villaverde recently investigated the occurrence (and origins of) parameters that are generically locally – but not globally – identifiable (so-called SLING parameters, short for “structurally locally identifiable but not globally”) (Barreiro and Villaverde 2023).

Our proofs, like those in several prior works (Ahmed et al. 2024; Bortner et al. 2024; Bortner et al. 2023; Bortner and Meshkat 2022, 2024; Chan et al. 2021; Gerberding et al. 2020; Gross et al. 2019, 2022), involve analyzing input-output equations, which are equations involving parameters, input variables, output variables, and their derivatives. This technique comes from following the differential-algebra approach to identifiability. We also rely on a recent combinatorial formula for coefficients of input-output equations (Bortner et al. 2023), which allows us to analyze whether (and, when possible, how) these coefficients can be used to recover some or all of the parameters.

This article is structured as follows. Section 2 provides background on linear compartmental models and their identifiability. Our results are proven in Section 3, and we conclude with a discussion in Section 4.

## Background

In this section, we introduce linear compartmental models (Section 2.1) and their input-output equations (Section 2.2). Subsequently, we define identifiability of models (Section 2.3) and of individual parameters (Section 2.4). Our notation matches that of prior work (Gerberding et al. 2020; Meshkat et al. 2015).

### Linear compartmental models

Informally, a linear compartmental model is a directed graph, with certain vertices (compartments) marked as “inputs” (representing inflows into the system), “outputs” (compartments at which experimental measurements can be taken), and “leaks” (outflows from the system). The formal definition follows.

#### Definition 2.1

A **linear compartmental model**, denoted by $(G, \textit{In}, \textit{Out}, \textit{Leak})$, consists of a directed graph $G = (V, E)$, in which each vertex $i\in V$ represents a **compartment** of the model, together with three sets $\textit{In}, \textit{Out}, \textit{Leak}\subseteq V$ which denote the sets of **input**, **output**, and **leak** compartments, respectively.

In a linear compartmental model $(G, \textit{In}, \textit{Out}, \textit{Leak})$, a directed edge $j\rightarrow i$ in *G* represents a flow from compartment-*j* to compartment-*i*. Each such edge has an associated parameter $k_{ij}$. Similarly, each leak compartment $p \in \textit{Leak}$ has an associated parameter, $k_{0 p}$. However, in this work, we focus on models without leaks (that is, $\textit{Leak}= \varnothing$).

**Fig. 2:**
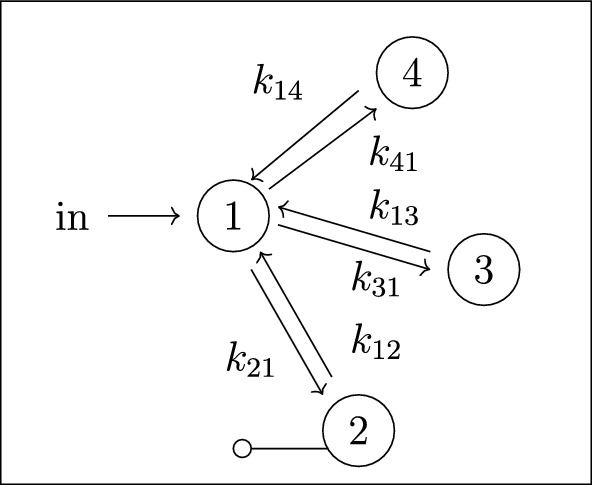
The mammillary model $\mathcal M = (G, \textit{In}, \textit{Out}, \textit{Leak})$ with 4 compartments and $\textit{In}=\{1\}$, $\textit{Out}=\{2\}$, and $\textit{Leak}= \varnothing$

In figures, we represent a linear compartmental model by its graph *G*, together with arrows for the inputs and the leaks, plus the following symbol for outputs: . See, for instance, Figures 1 and 2. The models depicted in both figures belong to an important class of linear compartmental models, called “mammillary models”, which is the focus of this work.

#### Definition 2.2

Let $\mathcal M = (G, \textit{In}, \textit{Out}, \textit{Leak})$ be a linear compartmental model.

1. $\mathcal M$ is a **mammillary model** if $G=(V,E)$ is a bidirected-star graph: $V=\{1,2,\dots , n\}$, where $n \ge 3$, and $E=\{ 1 \leftrightarrows 2, ~ 1 \leftrightarrows 3,~ \dots ,~ 1 \leftrightarrows n \}$.
2. ${\mathcal {M}}$ is **strongly connected** if *G* is strongly connected, that is, for each unordered pair of vertices *i*, *j* in *G*, there is a directed path in *G* from *i* to *j* and also a directed path from *j* to *i*.

Every mammillary model is strongly connected.

#### Definition 2.3

Let ${\mathcal {M}}=(G, \textit{In}, \textit{Out}, \textit{Leak})$ be a linear compartmental model with *n* compartments, where $G=(V,E)$. The **compartmental matrix** of ${\mathcal {M}}$ is the $n{\times }n$ matrix $A = (a_{ij})$ with entries as follows:

$$\begin{aligned} a_{ij} ~:=~ {\left\{ \begin{array}{ll} \displaystyle -k_{0i} - \sum _{\{ p \mid (i,p) \in E \} } k_{pi} & \text {if }i=j\text { and }i \in \textit{Leak},\\ \displaystyle - \sum _{ \{ p \mid (i,p) \in E \}} k_{pi} & \text {if }i=j\text { and }i \notin \textit{Leak},\\ k_{ij} & \text {if }i\ne j\text { and }j \rightarrow i\text { is an edge of }G,\\ 0 & \text {if }i\ne j\text { and }j\rightarrow i\text { is not an edge of }G. \end{array}\right. }. \end{aligned}$$

#### Example 2.4

The mammillary model in Figure 2 has the following compartmental matrix:

1 $$\begin{aligned} A ~=~\left[ \begin{array}{ccccc} -k_{21}-k_{31}-k_{41} & k_{12} & k_{13} & k_{14} \\ k_{21} & -k_{12} & 0 & 0\\ k_{31} & 0 & -k_{13} & 0\\ k_{41} & 0 & 0 & -k_{14} \end{array}\right] ~. \end{aligned}$$

Next, we describe how to use the compartmental matrix to write the ordinary differential equation (ODE) system arising from a model $\mathcal M = (G, \textit{In}, \textit{Out}, \textit{Leak})$. Let *n* denote the number of compartments, and let $x(t) = (x_1(t), x_2(t), \ldots , x_n(t))^{T}$ denote the vector of concentrations of all compartments at time *t*. For $i \in \textit{In}$, let $u_{i}(t)$ denote the inflow rate into compartment-*i* at time *t*. For $i \in \textit{Out}$, let $y_i(t)$ denote the corresponding output variable (measurement) at time *t*. Now $\mathcal M$ defines the following ODE system with inputs, where *A* is the compartmental matrix of $\mathcal M$:

2 $$\begin{aligned} x'(t)&~=~ Ax(t) + u(t)~, \nonumber \\ y_i(t)&~=~ x_i(t)~, \ \text { for all } i \in \textit{Out}~, \end{aligned}$$

where $u_i(t):=0$ for all $i \not \in \textit{In}$.

#### Example 2.5

*(Example* 2.4*, continued)* Using the compartmental matrix in (1), the ODE system (2) for the model in Figure 2 is as follows:

$$\begin{aligned} x_1'(t)&~=~ (-k_{21}-k_{31}-k_{41}) x_1 + k_{12} x_2 + k_{13} x_3 + k_{14} x_4 + u_1(t) \\ x_2'(t)&~=~ k_{21} x_1 -k_{12} x_2 \\ x_3'(t)&~=~ k_{31} x_1 -k_{13} x_3\\ x_4'(t)&~=~ k_{41} x_1 -k_{14} x_4 \\ y_2(t)&~=~ x_2(t)~. \end{aligned}$$

### Input-output equations

An **input-output equation** is an equation that holds along every solution *x*(*t*) of the ODEs (2) and involves only the parameters $k_{ij}$, input variables $u_{\ell }$, output variables $y_m$, and their derivatives. The next result is a formula for input-output equations, which is due to (Meshkat et al. 2015, Theorem 2).

#### Notation 2.6

We let $B^{j,i}$ denote the submatrix obtained from a matrix *B* by removing row-*j* and column-*i*.

#### Proposition 2.7

(Input-output equations (Meshkat et al. 2015)) Let ${\mathcal {M}} = (G, \textit{In}, \textit{Out}, \textit{Leak})$ be a linear compartmental model with *n* compartments and at least one input. Let *A* be the compartmental matrix. For $i\in \textit{Out}$, the following equation is an input-output equation for ${\mathcal {M}}$:

3 $$\begin{aligned} \det (\partial I-A)y_{i} ~=~ \sum _{j\in In}(-1)^{i+j}\det \left[ (\partial I-A)^{j,i}\right] u_j~, \end{aligned}$$

where $\partial I$ denotes the $n{\times }n$ matrix in which each diagonal entry is the differential operator *d*/*dt* and all off-diagonal entries are 0.

#### Example 2.8

*(Example* 2.5*, continued)* Recall that the model in Figure 2 has $\textit{In}=\{1\}$ and $\textit{Out}=\{2\}$. We use the compartmental matrix in (1) to obtain the input-output equation (3):

$$\begin{aligned} &  \det \left( \begin{bmatrix} d/dt+ k_{21}+k_{31}+k_{41} & -k_{12} & -k_{13} & -k_{14} \\ -k_{21} & d/dt +k_{12} & 0 & 0\\ -k_{31} & 0 & d/dt +k_{13} & 0\\ -k_{41} & 0 & 0 & d/dt+k_{14} \end{bmatrix} \right) y_{2} \\ &  \quad ~=~ (-1)^{2+1}\det \left( \begin{bmatrix} -k_{21} & 0 & 0\\ -k_{31} & d/dt +k_{13} & 0\\ -k_{41} & 0 & d/dt+k_{14} \end{bmatrix} \right) u_1~, \end{aligned}$$

which expands as follows:

4 $$\begin{aligned} y_{2}^{(4)}&+ (k_{12}+k_{13} + k_{14} + k_{21} + k_{31} + k_{41})y_{2}^{(3)} \nonumber \\&+ (k_{12}k_{13} + k_{12}k_{14} + k_{12}k_{31} + k_{12}k_{41} + k_{13}k_{14} + k_{13}k_{21} + k_{13}k_{41}\nonumber \\&+ k_{14}k_{21} + k_{14}k_{31})y_{2}^{(2)} \nonumber \\&+ (k_{12}k_{13}k_{14} + k_{12}k_{13}k_{41} + k_{12}k_{14}k_{31} + k_{13}k_{14}k_{21})y_{2}^{(1)} \nonumber \\&\quad \quad \quad ~=~ k_{21} u_{1}^{(2)} + (k_{13}k_{21} + k_{14}k_{21})u_{1}^{(1)} + k_{13}k_{14}k_{21} u_{1} \end{aligned}$$

Next, we introduce notation for an index set for all parameters of a model ${\mathcal {M}} = (G, \textit{In}, \textit{Out}, \textit{Leak})$, where $G=(V,E)$:

5 $$\begin{aligned} {\mathcal {P}}_{{\mathcal {M}}} ~:=~ \{ (j,i) \mid (j,i) \in E~,~ \textrm{or}~i=0~\textrm{and}~ j\in \textit{Leak}\}~. \end{aligned}$$

We use this set, which has size $|E|+ |\textit{Leak}|$, together with the input-output equation(s) (3), to define the **coefficient map** of $\mathcal M$:

$$\begin{aligned}\textsf{C}: {\mathbb {R}}^{ |E|+ |\textit{Leak}|} \rightarrow {\mathbb {R}} ^m~,\end{aligned}$$

which evaluates each vector of parameters $(k_{ij})_{(j,i) \in {\mathcal {P}}_{{\mathcal {M}}}}$ at the vector of non-constant coefficients of the input-output equation(s). (Here, *m* denotes the number of such coefficients.)

#### Example 2.9

*(Example* 2.8*, continued)* For the model in Figure 2, we use the input-output equation (4) to obtain the coefficient map $\textsf{C}: {\mathbb {R}}^6 \rightarrow {\mathbb {R}}^6$, where

6 $$\begin{aligned} \textsf{C}_1&:= k_{12}+k_{13} + k_{14} + k_{21} + k_{31} + k_{41} \nonumber \\ \textsf{C}_2&:= k_{12}k_{13} + k_{12}k_{14} + k_{12}k_{31} + k_{12}k_{41} + k_{13}k_{14} \nonumber \\&+ k_{13}k_{21} + k_{13}k_{41} + k_{14}k_{21} + k_{14}k_{31}\nonumber \\ \textsf{C}_3&:= k_{12}k_{13}k_{14} + k_{12}k_{13}k_{41} + k_{12}k_{14}k_{31} + k_{13}k_{14}k_{21} \nonumber \\ \textsf{C}_4&:= k_{21}\nonumber \\ \textsf{C}_5&:= k_{13}k_{21} + k_{14}k_{21}\nonumber \\ \textsf{C}_6&:=~ k_{13}k_{14}k_{21}~. \end{aligned}$$

The next result, Proposition 2.11 below, is used to analyze coefficient maps. We first need the following definition.

#### Definition 2.10

Let *H* be a directed graph.

1. A **spanning subgraph** is a subgraph of *H* that includes all vertices of *H*.
2. A **spanning incoming forest** of *H* is a spanning subgraph such that:
  each node has at most one outgoing edge, and
  the underlying undirected multigraph is a forest, that is, has no cycles.

Bortner *et al.* gave a combinatorial formula, which is in terms of spanning incoming forests, for the coefficients of input-output equations (Bortner et al. 2023, Theorem 3.1). We state the version of their result for the case of models with one input, one output, and no leaks (which is the situation we focus on in this work), as follows.

#### Proposition 2.11

(Coefficients of input-output equations for linear compartmental models (Bortner et al. 2023)) Let $n \ge 3$. Let ${\mathcal {M}} = (G, \textit{In}, \textit{Out}, \textit{Leak})$ be an *n*-compartment model with one input, one output, and no leaks: $\textit{In}=\{j\}$, $\textit{Out}= \{i\}$, and $\textit{Leak}= \varnothing$. Write the input-output equation (3) as follows:

7 $$\begin{aligned} y_i^{(n)} + c_{n-1} y_i^{(n-1)} + \dots + c_1 y_i' + c_0 y_i ~=~ d_{n-1} u_j^{(n-1)} + \dots + d_{1} u_j' + d_{0} u_j ~. \end{aligned}$$

Then the coefficients of the input-output equation (7) are given by:

8 $$\begin{aligned} c_k ~&=~ \sum _{F \in {\mathcal {F}}_{n-k}(G)} \pi _F \quad \text { for } k=0,1, \ldots , n-1~, \ \text { and } \nonumber \\ d_k ~&=~ \sum _{F \in {\mathcal {F}}^{j,i}_{n-k-1}(G^*_i)} \pi _F \quad \text { for } k=0,1,\ldots , n-1~, \end{aligned}$$

where:

- $G^*_i$ is the directed graph obtained from *G* by removing all outgoing edges from vertex-*i* (the output),
- ${\mathcal {F}}_{\ell } ( G )$ is the set of all spanning incoming forests of *G* with exactly $\ell$ edges,
- ${\mathcal {F}}_{\ell }^{j, i } ( G_i^* )$ is the set of all spanning incoming forests of $G_i^*$ with exactly $\ell$ edges, such that some connected component (of the underlying undirected graph) contains both of the vertices *j* and *i*,
- $\pi _F$ is the product of edge labels of a graph *F*, that is, $\pi _F:= \prod _{ e \in E_F } L(e)~$, where *L*(*e*) is the label of edge *e*, and $E_F$ is set of edges of *F*. If $E_F= \varnothing$, then $\pi _F:=1$.

#### Remark 2.12

($c_0=d_{n-1}=0$) In the context of Proposition 2.11, we have $c_0=0$. To see this, observe that the formula for $c_0$ in (8) is a sum over certain *n*-edge cycle-free subgraphs of *G*. However, *G* has only *n* vertices, and hence has no *n*-edge cycle-free subgraphs. We conclude that $c_0$ is the empty sum and hence is 0. An alternate proof can be given directly from (3). Similarly, $d_{n-1}=0$.

#### Remark 2.13

In Proposition 2.11, the input-output equation (7) corrects a sign error in (Bortner et al. 2023, Theorem 3.1), which erroneously contains a factor of $(-1)^{i+j}$ on the right-hand side.

#### Example 2.14

**Fig. 3:**
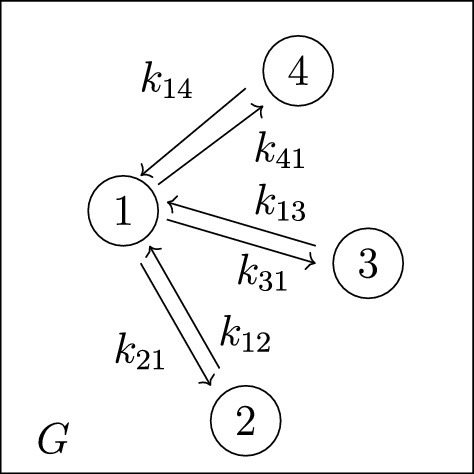
The graph *G* for the mammillary model in Figure 2

**Fig. 4:**
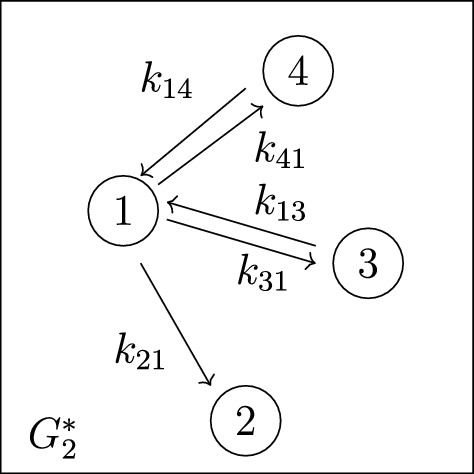
The graph $G_{2}^*$, obtained from the graph *G* in Figure 3 by removing the outgoing edge from compartment-2 (the output in Figure 2)

**Fig. 5:**
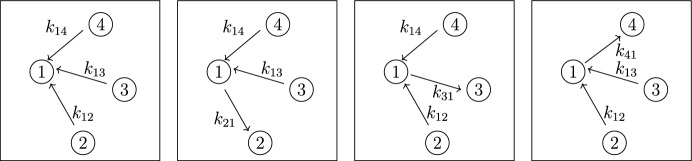
Spanning incoming forests of *G* with 3 edges.

**Fig. 6:**
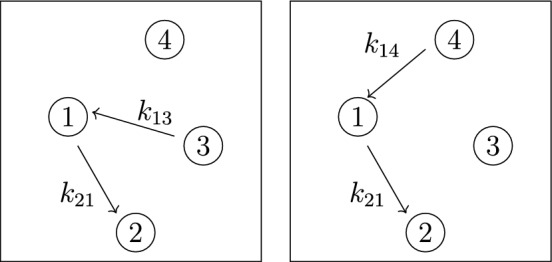
Spanning incoming forests of $G_2^*$ with 2 edges in which compartments 1 and 2 are connected

*(Example* 2.9*, continued)* Recall that the 4-compartment mammillary model $\mathcal M = (G, \textit{In}, \textit{Out}, \textit{Leak})$ in Figure 2 has $\textit{In}=\{1\}$, $\textit{Out}=\{2\}$, and $\textit{Leak}= \varnothing$. Following the notation in (7), the coefficients $c_i$ and $d_i$ are given by $(c_3, c_2, c_1, d_2, d_1, d_0):= (\textsf{C}_1, \textsf{C}_2, \textsf{C}_3, \textsf{C}_4, \textsf{C}_5, \textsf{C}_6)$, where the $\textsf{C}_i$ are as in (6), and also $c_0=0$. Next, the graphs *G* and $G_2^*$ are shown in Figures 3 and 4, respectively. It is now straightforward to check that the coefficients $c_i$ and $d_i$ satisfy the formulas (8). For instance, $c_1=\textsf{C}_3$ is a sum over the 4 spanning incoming forests of *G* with $4-1=3$ edges; these 4 forests are depicted in Figure  5. Similarly, $d_1= \textsf{C}_5$ is a sum over the 2 spanning incoming forests of $G_2^*$ with $4-1-1=2$ edges in which compartments 1 and 2 are connected; these 2 forests are shown in Figure  6.

### Identifiability of models

The idea behind the problem of model identifiability is as follows: Given measured values of how much enters and exits the input and output compartments, respectively, can we infer all parameter values of a model? To be more precise, we are asking whether, from generic values of the inputs and initial conditions, we can recover the parameters (at least locally, which means up to a finite set) from exact measurements of the inputs and the outputs. This is the concept of *generic local identifiability*.

This notion of identifiability is equivalent (for strongly connected models with at least one input) to Definition 2.15 below, which checks identifiability by way of coefficient maps. This equivalence was proven by  (Ovchinnikov et al. 2023, Main Result 3).

In what follows, we use “almost every” in the usual measure-theoretic sense: “**almost every** *x* in a set *X*” means “for all *x* in $X \smallsetminus Z$, for some measure-zero subset *Z* of *X*”.

#### Definition 2.15

*(Identifiability of *${\mathcal {M}}$*)* Let $\mathcal M =(G, \textit{In}, \textit{Out}, \textit{Leak})$ be a linear compartmental model that is strongly connected and has at least one input. Let $G=(V,E)$, and let $\textsf{C}: \mathbb {R}^{|E| + |\textit{Leak}|} \rightarrow \mathbb {R}^m$ denote the coefficient map of $\mathcal M$.

1. $\mathcal M$ is **generically locally identifiable** if almost every point in $\mathbb {R}^{|E| + |\textit{Leak}|}$ has an open neighborhood on which $\textsf{C}$ is injective.
2. $\mathcal M$ is **unidentifiable** if $\mathcal M$ is not generically locally identifiable.

The following result, which was proved by (Bortner et al. 2023, Corollary 5.4), characterizes identifiability of mammillary models (Definition 2.2(1)) with one input and one output.

#### Proposition 2.16

(Identifiability of mammillary models) A mammillary model $\mathcal M =(G, \textit{In}, \textit{Out}, \textit{Leak})$ with $|\textit{In}|=|\textit{Out}|=1$ is generically locally identifiable if and only if $|Leak| \le 1$ and one or more of the following hold: (1) $In=Out$, (2) $In=\{1\}$, or (3) $Out=\{1\}$.

#### Example 2.17

*(Example* 2.14*, continued)* For the mammillary model in Figure 2, we have $In = \{1\}$, $\textit{Out}=\{2\}$, and no leaks. Therefore, by Proposition 2.16, the model is generically locally identifiable.

In the next subsection, we examine whether the individual parameters of this model are generically globally identifiable versus generically locally identifiable.

### Identifiability of parameters

Definition 2.15 above concerns the identifiability of models, whereas the next definition pertains to the identifiability of individual parameters of a model (cf. Hong et al. 2018, Definition 2.5).

#### Definition 2.18

*(Identifiability of parameter *$k_{pq}$*)* Let $\mathcal M =(G, \textit{In}, \textit{Out}, \textit{Leak})$ be a strongly connected, linear compartmental model with at least one input. Let $G=(V,E)$, and let $\textsf{C}: \mathbb {R}^{|E| + |\textit{Leak}|} \rightarrow \mathbb {R}^m$ denote the coefficient map of $\mathcal M$. Let $k_{pq}$ be a parameter of $\mathcal M$, i.e., (*q*, *p*) is in the index set ${\mathcal {P}}_{{\mathcal {M}}}$, in (5). Write each parameter vector $(k_{i j})_{(j,i) \in {\mathcal {P}}_{{\mathcal {M}}}}$ as $( k_{pq};~ {\widetilde{k}} )$, where ${\widetilde{k}}:= (k_{ij})_{(j,i) \in {\mathcal {P}}_{{\mathcal {M}}} \smallsetminus \{ (q,p)\}}$; this allows us to write the coefficient map as $\textsf{C}= \textsf{C}( k_{pq};~ {\widetilde{k}})$.

The parameter $k_{pq}$ is:

1. **globally identifiable** if, for every $( k^*_{pq};~ {\widetilde{k}}^* )$ in ${\mathbb {R}}^{|E| + |\textit{Leak}|}$, the following set (which is a projection of a preimage of $\textsf{C}( k^*_{pq};~ {\widetilde{k}}^*)$) has size one:
  9 $$\begin{aligned} \{ k_{pq} \mid \mathrm {there~exists~} \widetilde{k} \in {\mathbb {R}}^{|E| + |\textit{Leak}|} \mathrm {~such~that~} \textsf{C}( k_{pq};~ {\widetilde{k}}) = \textsf{C}( k^*_{pq};~ {\widetilde{k}}^*) \}~; \end{aligned}$$
2. **generically globally identifiable** if, for almost every $( k^*_{pq};~ {\widetilde{k}}^* )$ in ${\mathbb {R}}^{|E| + |\textit{Leak}|}$, the set (9) has size one;
3. **generically locally identifiable** if, for almost every $( k^*_{pq};~ {\widetilde{k}}^* )$ in ${\mathbb {R}}^{|E| + |\textit{Leak}|}$, the set (9) is finite; and
4. **unidentifiable** if $k_{pq}$ is not generically locally identifiable.

#### Notation 2.19

Following Barreiro and Villaverde, we use **SLING** (which stands for “structurally locally identifiable but not globally”) to refer to a parameter that is generically locally identifiable, but *not* generically globally identifiable (Barreiro and Villaverde 2023).

#### Remark 2.20

(Identifiability of models versus parameters) A model is generically locally identifiable (Definition 2.15(1)) if and only if all of its parameters are generically locally identifiable (Definition 2.18(3)); cf. (Hong et al. 2018, Remark 2.6(a)). Equivalently, a model is unidentifiable if and only if at least one of its parameters is unidentifiable.

#### Remark 2.21

(Software for checking identifiability) Identifiability of parameters can be checked using software. One option is the Maple software SIAN (Structural Identifiability ANalyser) (Hong et al. 2019), which is based on theory developed by Hong et al. (2018). (This theory is developed over ${\mathbb {C}}$, whereas we work over ${\mathbb {R}}$; hence, users of SIAN need to be careful when interpreting the output.) Another option is the Julia package StructuralIdentifiability.jl, which implements theory of Dong et al. (2023).

#### Remark 2.22

(Restricting parameter values) A meaningful extension of Definition 2.18 would be to include, as part of the setup, a subset of the full parameter space ${\mathbb {R}}^{|E| + |Leak|}$ (or a collection of subsets of ${\mathbb {R}}$, one for each parameter). The reason would be to allow a researcher to take into account known information about parameter values (positivity, for instance, or restriction to an interval). Here, however, for simplicity, we do not incorporate this addition.

#### Identifiable functions

One of our primary methods for proving that certain parameters are generically globally identifiable or generically locally identifiable is through the theory of “identifiable functions” (see Definition 2.23 and Proposition 2.25 below). This approach makes precise the idea that identifiable parameters are the ones that we can “solve for” in terms of the coordinates of the coefficient map.

We follow the notation of Meshkat et al. (2018) in what follows.

##### Definition 2.23

Let $\textsf{C}:{\mathbb {R}}^{|E|+ |\textit{Leak}|} \rightarrow {\mathbb {R}}^m$ be the coefficient map of a linear compartmental model, and let $f:{\mathbb {R}}^{|E|+ |\textit{Leak}|} \rightarrow {\mathbb {R}}$ be some other function. The function *f* is:

- an **identifiable function** from $\textsf{C}$ if, for all $p,p'\in {\mathbb {R}}^{|E|+ |\textit{Leak}|}$, we have that $\textsf{C}(p)=\textsf{C}(p')$ implies that $f(p)=f(p')$.
- a **generically identifiable function** from $\textsf{C}$ if *f* is identifiable from $\textsf{C}$ on some open, dense subset $U\subseteq {\mathbb {R}}^{|E|+ |\textit{Leak}|}$.
- a **locally identifiable function** from $\textsf{C}$ if there is an open, dense subset $U\subseteq {\mathbb {R}}^{|E|+ |\textit{Leak}|}$ such that, for all $p\in U$, there is an open neighborhood $U_p$ of *p* such that *f* is identifiable from $\textsf{C}$ on $U_p$.

##### Remark 2.24

(Identifiable parameters and functions) The relationship between Definitions 2.18 and 2.23 is as follows. For a parameter $k_{ij}$ of a model with coefficient map $\textsf{C}$, if the function $f=k_{ij}$ is identifiable (respectively, generically identifiable or locally identifiable), then the parameter $k_{ij}$ is globally identifiable (respectively, generically globally identifiable or generically locally identifiable).

The following proposition, which is similar in spirit to (Meshkat et al. 2018, Proposition 4.4) (and can be viewed as an instantiation of the ideas in (Hong et al. 2018, Proposition 3.4) and (Ovchinnikov et al. 2022, Definition 2.5)), will help us prove identifiability properties of parameters:

##### Proposition 2.25

Let $\mathcal M =(G, \textit{In}, \textit{Out}, \textit{Leak})$ be a linear compartmental model with coefficient map $\textsf{C}: \mathbb {R}^{|E| + |\textit{Leak}|} \rightarrow \mathbb {R}^m$. Let $k_{ij}$ be a parameter of ${\mathcal {M}}$. Suppose that there is a function $g: \mathbb {R}\times \mathbb {R}^{|E| + |\textit{Leak}|} \rightarrow \mathbb {R}$, which we write as $g = g(z;(k_{i j})_{(j,i) \in {\mathcal {P}}_{{\mathcal {M}}}})$, for which:

1. for some $d\ge 1$, there exist polynomial functions $q_0,q_1,\dots , q_d: \mathbb {R}^{|E| + |\textit{Leak}|} \rightarrow \mathbb {R}$ that are identifiable from $\textsf{C}$, such that
  $$\begin{aligned} g ~=~ q_d z^d + q_{d-1}z^{d-1} + \dots + q_0~, \end{aligned}$$
  and the leading coefficient $q_d$ is not the zero function; and
2. the function $g|_{z=k_{ij}}: \mathbb {R}^{|E| + |\textit{Leak}|} \rightarrow \mathbb {R}$ is the zero function.

Then the parameter $k_{ij}$ is generically locally identifiable. Moreover, if $d=1$, then the parameter $k_{ij}$ is generically globally identifiable.

##### Proof

Let $g,~q_0,q_1,\dots , q_d$, and $k_{ij}$ be as in the statement of the proposition. The set $U:= \mathbb {R}^{|E| + |\textit{Leak}|} \smallsetminus \{q_d=0\}$ is an open, dense subset of $\mathbb {R}^{|E| + |\textit{Leak}|}$ (here we use the fact that the polynomial $q_d$ is nonzero). In particular, the complement of *U* has measure zero.

Fix $$(k^*_{ij}; \widetilde{k}^*) \in {U}$$ (here, the notation $$(k^*_{ij}; \widetilde{k}^*)$$ is like that in Definition 2.18). By Definition 2.18(2), it suffices to show that there are only finitely many values $$k^{**}_{ij}$$ for which there exist some $$ \widetilde{k}^{**} \in \mathbb {R}^{|E| + |\textit{Leak}|-1}$$ with $$ \textsf{C}(k^{**}_{ij}; \widetilde{k}^{**}) = \textsf{C}(k^*_{ij}; \widetilde{k}^*)$$.

To this end, recall that $$q_0,q_1,\dots , q_d$$ are identifiable from $$\textsf{C}$$. This fact implies that the following holds for all $$i=0,1,\dots ,d$$, whenever $$ \textsf{C}(k^{**}_{ij}; \widetilde{k}^{**}) = \textsf{C}(k^*_{ij}; \widetilde{k}^*)$$:

$$\begin{aligned} q_i^* ~&:=~ q_i|_{(k_{ij}; \widetilde{k})=(k^*_{ij}; \widetilde{k}^*)} ~=~ q_i|_{(k_{ij}; \widetilde{k})=(k^{**}_{ij}; \widetilde{k}^{**)}}~. \end{aligned}$$

Now recall that $$g|_{z=k_{ij}}$$ is the zero function (by assumption). It now follows that, if $$ \textsf{C}(k^{**}_{ij}; \widetilde{k}^{**}) = \textsf{C}(k^*_{ij}; \widetilde{k}^*)$$, then $$k^{**}_{ij}$$ is a root of the following univariate polynomial (which is nonzero because we constructed *U* to be disjoint from $$\{q_d = 0\}$$):

$$\begin{aligned} g|_{(k_{ij}; \widetilde{k})=(k^*_{ij}; \widetilde{k}^*)} ~=~ q^*_d z^d + q^*_{d-1}z^{d-1} + \dots + q^*_0~. \end{aligned}$$

Thus, $$k^{**}_{ij}$$ can take at most *d* values, and so we conclude that the parameter $$k_{ij}$$ is generically locally identifiable. Additionally, if $$d=1$$, then there is only one possible value (namely, $$k^{**}_{ij} = k^{*}_{ij}$$) and so, in this case, the parameter $$k_{ij}$$ is generically globally identifiable. $$\square $$

##### Example 2.26

*(Example* 2.17*, continued)* We return to the model in Figure 2 and its coefficient map (6). We saw that $\textsf{C}_4= k_{21}$, and so the parameter $k_{21}$ is easily recovered from the coefficients $\textsf{C}_i$. More precisely, $k_{21}$ is an identifiable function from $\textsf{C}$, so Remark 2.24 implies that $k_{21}$ is globally identifiable. Alternatively, we can draw a slightly weaker conclusion by noting that $k_{21}$ satisfies the hypotheses of Proposition 2.25 with $g:=-k_{21}+z$ (so, $q_0=-k_{21}$ and $q_1=1$), and hence $k_{21}$ is generically globally identifiable. It turns out that no other parameter of this model is generically globally identifiable; in fact, all remaining parameters are SLING. This fact can be proven directly or by examining the symmetry in the model (see Example 2.29 below).

In Example 2.26, we saw that one of the coefficients (namely, $\textsf{C}_4$) equals the parameter $k_{21}$. This parameter corresponds to the edge from the input (at compartment-1) to the output (at compartment-2). This observation generalizes, as follows.

##### Lemma 2.27

(Global identifiability of edge from input to output) Let $\mathcal M =(G, \textit{In}, \textit{Out}, \textit{Leak})$ be a strongly connected, linear compartmental model with $\textit{In}=\{i\}$, $\textit{Out}=\{j\}$, and $\textit{Leak}= \varnothing$. If $i \rightarrow j$ is an edge of *G* (with parameter $k_{ji}$), then $k_{ji}$ is globally identifiable.

##### Proof

Proposition 2.11 implies that one of the coefficients of the input-output equations, namely, $d_{n-2}$, as in (7), equals $k_{ji}$. Now the result follows from Remark 2.24. $\square$

##### Remark 2.28

Gogishvili constructed a large database of linear compartmental models, and observed that Lemma 2.27 holds for all models in the database (Gogishvili 2024, §5).

#### Symmetric edge parameters

We end this section by asserting that edges that are symmetric share the same identifiability properties (Lemma 2.32 below). This simple idea has appeared in prior work (e.g., Gogishvili 2024, §2.2 and Cobelli et al. (1979)), and we motivate this result through the following example.

##### Example 2.29

*(Example* 2.26*, continued)* Observe in Figure 2 the symmetry between compartments 3 and 4. This symmetry tells us that the parameters for edges incident to 3 (namely, $k_{13}$ and $k_{31}$) and those incident to 4 (namely, $k_{14}$ and $k_{41}$) are not generically globally identifiable, because their values are indistinguishable from those of their symmetric pair. This indistinguishability also can be seen in the coefficients (6) of the input-output equation: Each coefficient is invariant under exchanging $k_{13}$ and $k_{14}$ with $k_{31}$ and $k_{41}$, respectively. We make these ideas precise through the next definition and lemma.

##### Definition 2.30

Let *n* be a positive integer.

1. An **automorphism** of a directed graph $G=(V,E)$, where $V=\{1,2,\dots , n\}$, is a permutation $\sigma$ of *V* such that $\sigma (G):= (V, \sigma (E)) = G$, where $\sigma (E):= \{\sigma (e) \mid e \in E \}$ and $\sigma (e):= (\sigma (i),\sigma (j))$, if $e=(i,j) \in E$.
2. For linear compartmental models $\mathcal M =(G, \textit{In}, \textit{Out}, \textit{Leak})$ and $\mathcal M' =(G', \textit{In}', \textit{Out}', \textit{Leak}')$ with $G=G'$, a **morphism** from $\mathcal M$ to $\mathcal M'$ is an automorphism $\sigma$ of *G* such that $\sigma (\textit{In}) = \textit{In}'$, $\sigma (\textit{Out}) = \textit{Out}',$ and $\sigma (\textit{Leak})=\textit{Leak}'$.
3. An **automorphism** of a linear compartmental model $\mathcal M$ is a morphism from $\mathcal M$ to $\mathcal M$.

##### Remark 2.31

(Input-output equations under morphisms) Given a morphism from $\mathcal M$ to $\mathcal M'$, it is straightforward to see from definitions that the input-output equations (3) for $\mathcal M'$ are obtained from the input-output equations for $\mathcal M$ by permuting the subscripts according to $\sigma$ (i.e., we replace $k_{ij}$ and $k_{0j}$ by, respectively, $k_{\sigma (i),\sigma (j)}$ and $k_{0 \sigma (j)}$, for relevant indices *i*, *j*).

##### Lemma 2.32

(Symmetric edge parameters are SLING or unidentifiable) Consider a linear compartmental model $\mathcal M =(G, \textit{In}, \textit{Out}, \textit{Leak})$, where $G=(V,E)$. Consider two edges, $e=(q,p) \in E$ and $e'=(q',p') \in E$, with $e \ne e'$. If there exists an automorphism $\sigma$ of ${\mathcal {M}}$ such that $\sigma (e)= e'$, then the parameters for *e* and $e'$ (namely, $k_{pq}$ and $k_{p'q'}$) are either both SLING parameters or both unidentifiable parameters of ${\mathcal {M}}$.

##### Proof

Let ${\mathcal {M}}$ be a linear compartmental model, with automorphism $\sigma$ and edges $e=(q,p)$ and $e'=(q',p')$, as in the statement of the lemma. Our first aim is to show that the parameters $k_{pq}$ and $k_{p'q'}$ have the same identifiability properties (as in Definition 2.18). Accordingly, consider any parameter vector ${\textbf{k}}^* \in {\mathbb {R}}^{|E| + |\textit{Leak}|}$. It suffices to show that the projected set (9) for the edge $e=(q,p)$ is equal to the corresponding projected set for $e'=(q',p')$. In fact, we need only show one containment, as the reverse containment will follow by applying the same argument to the inverse morphism $\sigma ^{-1}$.

Let $\textsf{C}:{\mathbb {R}}^{|E|+|\textit{Leak}|} \rightarrow {\mathbb {R}}^m$ denote the coefficient map of ${\mathcal {M}}$, and let $\pi _{pq}: {\mathbb {R}}^{|E|+|\textit{Leak}|} \rightarrow {\mathbb {R}}$ (respectively, $\pi _{p'q'}$) denote the projection onto the coordinate $k_{pq}$ (respectively, $k_{p'q'}$). Now consider the following diagram, where $\widetilde{\sigma }$ permutes the coordinates according to $\sigma$ (the coordinates $k_{ij}$ and $k_{0j}$ are sent to, respectively, $k_{\sigma (i),\sigma (j)}$ and $k_{0 \sigma (j)}$, for relevant indices *i*, *j*): 
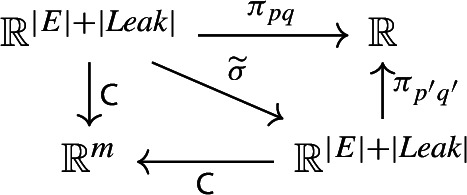


In this diagram, the upper triangle commutes by construction. The lower triangle also commutes, by Remark 2.31 and the fact that $\sigma$ is an automorphism.

We claim that the fact that the diagram above commutes, implies that the projected set (9) for *e*, namely, $\pi _{pq}(\textsf{C}^{-1}(\textsf{C}({\textbf{k}}^*)))$ is contained in the projected set for $e'$, namely, $\pi _{p'q'}(\textsf{C}^{-1}(\textsf{C}({\textbf{k}}^*)))$. Indeed, this is a straightforward “diagram chasing” argument, which we outline as follows. Given $x \in \pi _{pq}(\textsf{C}^{-1}(\textsf{C}({\textbf{k}}^*)))$, let $\overline{y}:= \widetilde{\sigma }(\overline{x})$, where $\pi _{pq}(\overline{x})=x$ and $\overline{x} \in \textsf{C}^{-1}(\textsf{C}({\textbf{k}}^*))$. The lower commutative triangle is used to show that $\overline{y} \in \textsf{C}^{-1}(\textsf{C}({\textbf{k}}^*))$, while the upper commutative triangle is used to show that $\pi _{p'q'}(\overline{y})=x$. Hence, $x \in \pi _{p'q'}(\textsf{C}^{-1}(\textsf{C}({\textbf{k}}^*)))$.

To complete the proof, we need only show that the parameter $k_{pq}$ is not generically globally identifiable. Accordingly, let *Z* denote the hyperplane in ${\mathbb {R}}^{|E| + |\textit{Leak}|}$ defined by $k_{pq} = k_{p'q'}$ (so, *Z* is a measure-zero subset of ${\mathbb {R}}^{|E| + |\textit{Leak}|}$). Let ${\textbf{k}}^*:= (k^*_{i j})_{(j,i) \in {\mathcal {P}}_{{\mathcal {M}}}} \in {\mathbb {R}}^{|E| + |\textit{Leak}|} \smallsetminus Z$. Consider $\widetilde{\sigma }({\textbf{k}}^*) = (k^*_{\sigma (i) \sigma (j)})_{(j,i) \in {\mathcal {P}}_{{\mathcal {M}}}}$, where $\sigma (0):=0$ (to account for leak parameters $k^*_{0j}$). From the commutative diagram shown earlier in the proof, we have $\textsf{C}({\textbf{k}}^*) = \textsf{C}(\widetilde{\sigma }({\textbf{k}}^*))$. Hence, the projected set (9) for *e*, contains both $k^*_{pq}$ and $k^*_{ \sigma (p) \sigma (q)}$ (which are distinct, because ${\textbf{k}}^* \notin Z$). We conclude that, as desired, $k_{qp}$ is not generically globally identifiable. $\square$

## Results

In this section, we investigate the identifiability of all parameters in mammillary models with one input, one output, and no leaks. For ease of notation, we let ${\mathcal {M}}_n(i,j)$ denote the *n*-compartment mammillary model $(G, \textit{In}, \textit{Out}, \textit{Leak})$ with $\textit{In}=\{i\}$, $\textit{Out}=\{j\}$, and $\textit{Leak}=\varnothing$. For instance, ${\mathcal {M}}_4(1,2)$ denotes the model shown earlier in Figure 2.

Up to symmetry, the mammillary models ${\mathcal {M}}_n(i,j)$ fall into five families. These families are defined by the location of the input and the output, so we consider the following representatives, one for each of the five families:

10 $$\begin{aligned} {\mathcal {M}}_n(1,1),~ {\mathcal {M}}_n(1,2), ~{\mathcal {M}}_n(2,1),~ {\mathcal {M}}_n(2,2),~ \textrm{and}~ {\mathcal {M}}_n(2,3)~. \end{aligned}$$

### Remark 3.1

Proposition 2.16 implies that, among the models (10), only the model ${\mathcal {M}}_n(2,3)$ is unidentifiable or, equivalently, by Remark 2.20, contains unidentifiable parameters. Hence, every parameter in the remaining four models in (10) is generically locally identifiable (and thus is SLING or generically globally identifiable).

Our main result, which was summarized earlier in Figure 1, is as follows.

### Theorem 3.2

(Main result) For mammillary models with one input, one output, and no leaks, the parameters have the following identifiability properties:

- For all $n \ge 3$, every parameter of ${\mathcal {M}}_n(1,1)$ is SLING.
- For all $n \ge 4$, every parameter of ${\mathcal {M}}_n(1,2)$ is SLING, except the parameter $k_{21}$ labeling the edge from the input to output, which is globally identifiable.
- For all $n \ge 4$, every parameter of ${\mathcal {M}}_n(2,1)$ is SLING, except the parameters $k_{12}$ and $k_{21}$ labeling the edges between the input and output, which are, respectively, globally identifiable and generically globally identifiable.
- For all $n \ge 4$, every parameter of ${\mathcal {M}}_n(2,2)$ is SLING, except the parameters $k_{12}$ and $k_{21}$ labeling the edges between the input and output, which are generically globally identifiable.
- For all $n \ge 5$, the parameters of ${\mathcal {M}}_n(2,3)$ that label the edges from non-input, non-output peripheral compartments to the central compartment (namely, $k_{14}, k_{15}, \dots , k_{1n}$) are SLING.

The remainder of this section is dedicated to proving Theorem 3.2 (see Propositions 3.9, 3.11, 3.12, 3.14, and 3.16 below). We also state a conjecture concerning model ${\mathcal {M}}_n(2,3)$ that, if proven, would elevate Theorem 3.2 to match what is depicted in Figure 1 (see Conjecture 3.18). This conjecture is supported by examples we computed using software (see Remark 2.21).

### Remark 3.3

In Theorem 3.2, we see each of the models ${\mathcal {M}}_n(1,2)$ and ${\mathcal {M}}_n(2,1)$ has one parameter that is globally identifiable (which is stronger than being generically globally identifiable). This global identifiability comes from the fact that such a parameter corresponds to the edge from input to output (recall Lemma 2.27).

### Preliminaries

All five model families (10) share the same compartmental matrix *A* (for a given number of compartments *n*). Thus, the set of coefficients on the left-hand side of the input-output equation (3) also is the same. Accordingly, we give a formula for these coefficients in this subsection (Proposition 3.7 below). Appearing in this formula (and other formulas for coefficients we see later in this section) are elementary symmetric polynomials.

#### Definition 3.4

Let *k* and *n* be positive integers, with $1 \le k \le n$. Let $X_n$ denote the set of variables $x_1, x_2,\ldots , x_n$. The *k*-th **elementary symmetric polynomial** on $X_n$, which we denote by $e_k(X_n)$, is given by

$$\begin{aligned} e_k(X_n) ~:=~ \sum _{\begin{array}{c} I \subseteq [n] \\ |I| = k \end{array}} \left( \prod _{i\in I} x_i\right) ~. \end{aligned}$$

We additionally adopt the following standard convention: $e_0(X_n):=1$.

#### Example 3.5

*(Example* 2.26*, continued)* We revisit the mammillary model ${\mathcal {M}}_4(1,2)$ in Figure 2. The left-hand side coefficients of the input-output equation (see (6)) can be rewritten in terms of elementary symmetric polynomials, as follows:

11 $$\begin{aligned} c_3 ~&=~ \textsf{C}_1 ~=~ e_1({\mathcal {I}}) ~+~ k_{21} + k_{31} + k_{41} ~, \nonumber \\ c_2 ~&=~ \textsf{C}_2 ~=~ e_2({\mathcal {I}}) ~+~ k_{21} e_1({\mathcal {I}} \smallsetminus \{k_{12}\} ) + k_{31} e_1({\mathcal {I}} \smallsetminus \{k_{13}\}) + k_{41} e_1({\mathcal {I}} \smallsetminus \{k_{14}\}) \nonumber \\ c_1 ~&=~ \textsf{C}_3 ~=~ e_3({\mathcal {I}}) ~+~ k_{21} e_2({\mathcal {I}} \smallsetminus \{k_{12}\} ) + k_{31} e_2({\mathcal {I}} \smallsetminus \{k_{13}\}) + k_{41} e_2({\mathcal {I}} \smallsetminus \{k_{14}\})~, \end{aligned}$$

where ${\mathcal {I}}:= \{k_{12},k_{13}, k_{14} \}$ is the set of all “incoming” edge labels (that is, labels of edges of the graph *G* in Figure 3 that are directed toward the central compartment).

**Fig. 7:**
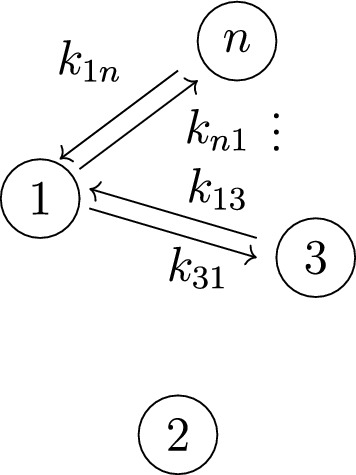
Graph obtained from the star graph in Figure 3 by removing the pair of edges between compartment-1 and compartment-2

Example 3.5 displayed formulas (11) for the left-hand side coefficients for ${\mathcal {M}}_4(1,2)$. We give a new formula for these coefficients, using subgraphs of the graph in Figure 7, as follows.

#### Example 3.6

*(Example* 3.5*, continued)* For the model ${\mathcal {M}}_4(1,2)$, the left-hand side coefficients of the input-output equation (shown in (6) and (11)) can be rewritten by grouping terms based on whether they contain $k_{21}$, contain $k_{12}$, or contain neither $k_{21}$ nor $k_{12}$:

12 $$\begin{aligned} c_3 ~&=~ k_{21} + k_{12} + g_1 \nonumber \\ c_{2} ~&=~ k_{21}~e_{1}(\Sigma ) + k_{12} g_1 + g_2 \nonumber \\ c_{1} ~&=~ k_{21}~e_{2}(\Sigma ) + k_{12} g_2~, \end{aligned}$$

where

$$\begin{aligned} \Sigma&:=\{k_{13},k_{14}\}~, \quad g_1~:=~k_{13} + k_{14} + k_{31} + k_{41}~, \quad \textrm{and}\\ g_2&:= k_{13}k_{14} + k_{13}k_{41} + k_{14}k_{31}~. \end{aligned}$$

Notice that $g_1$ is the sum over all edges in the $n=4$ version of the graph in Figure 7, and $g_2$ is the sum over pairs of edges in Figure 7 that do not form a cycle. Also, it is straightforward to deduce the following equation from the equations (12):

13 $$\begin{aligned} k_{12}^2 c_3 - k_{12} c_2 + c_1 ~=~ (k_{12}^2 - k_{12} e_1(\Sigma ) + e_2(\Sigma ) ) k_{21} + k_{12}^3~. \end{aligned}$$

Next, alternate formulas for $g_1$ and $g_2$ are given by:14The formulas (12) and equations (13)–(14) generalize to what is shown in (15), (17), and (18), respectively, in the next result.

#### Proposition 3.7

(Left-hand side coefficients) Let $n \ge 3$. Consider an *n*-compartment mammillary model ${\mathcal {M}} = (G, \textit{In}, \textit{Out}, \textit{Leak})$ with $|\textit{In}|= |\textit{Out}|= 1$ and $\textit{Leak}=\varnothing$. Let $c_0,c_1,\dots , c_{n-1}$ denote the coefficients of the left-hand side of the input-output equation, as in (7). Then the following hold:

- $c_0=0$, and the coefficients $c_1,c_2,\dots , c_{n-1}$ are given by the following formulas:
  15 $$\begin{aligned} c_{n-1} ~&=~ k_{21} + k_{12} g_0 + g_1\nonumber \\ c_{n-2} ~&=~ k_{21}~e_{1}(\Sigma ) + k_{12} g_1 + g_2\nonumber \\ c_{n-3} ~&=~ k_{21}~e_{2}(\Sigma ) + k_{12} g_2 + g_3\nonumber \\&~ \vdots \nonumber \\ c_{1} ~&=~ k_{21}~e_{n-2}(\Sigma ) + k_{12} g_{n-2} + g_{n-1} ~, \end{aligned}$$
  where $\Sigma :=\{k_{13}, k_{14}, \dots , k_{1n} \}$ and, for $i=0,1,2,\dots , n-1$, we denote:
  16 $$\begin{aligned} g_i ~:=~ \sum _{F \mathrm {~is~an~} i\mathrm {-edge~spanning,~incoming~forest~of~} \widetilde{G} } \pi _F~, \end{aligned}$$
  where $\widetilde{G}$ denotes the graph in Figure 7.
- The following equation holds:
  17 $$\begin{aligned}&k_{12}^{n-2} c_{n-1} - k_{12}^{n-3} c_{n-2} + k_{12}^{n-4} c_{n-3} - \dots \pm c_{1} \nonumber \\&\quad \quad ~=~ \left( k_{12}^{n-2} - k_{12}^{n-3}~ e_1(\Sigma ) + k_{12}^{n-4}~ e_2(\Sigma ) - \dots \pm e_{n-2}(\Sigma ) \right) k_{21} ~+~ k_{12}^{n-1}~. \end{aligned}$$
- The terms $g_i$ are given by $g_0=1$, $g_{n-1}=0$, and the following formula: 18 where $\Sigma :=\{k_{13},k_{14},\dots ,k_{1n}\}$ is the set of all “incoming” edge labels of $\widetilde{G}$ (the graph in Figure 7), and where *M* denotes the following $(n-2) \times (n-2)$ matrix: 19
- The determinant of this matrix *M* is, up to sign, the *Vandermonde polynomial* $\prod _{3 \le j < \ell \le n} (k_{1j}- k_{1 \ell })$.

#### Proof

The equality $c_0=0$ was given earlier (Remark 2.12). Next, for $i=1,2,\dots , n-1$, recall from Proposition 2.11 that the coefficient $c_i$ is the sum of all terms $\pi _F$, where *F* is a spanning incoming forest that is an $(n-i)$-edge subgraph of the star graph *G* (in Figure 3). Such a forest can not contain both of the edges $k_{21}$ and $k_{12}$ (to avoid a cycle), so each $c_i$ is a sum over three types of forests:

- $\underline{\hbox { forests involving }\,k_{21}\,\hbox { (but not }\,k_{12})}$ – in this case, as $k_{21}$ is outgoing from compartment-1, the remaining edges must be chosen from the “incoming” edges (those directed toward compartment-1) from $3, 4, \dots , n$ (and can be arbitrarily chosen); these edges are indexed by the set $\Sigma$;
- $\underline{\hbox { forests involving }\,k_{12}\,\hbox { (but not }\,k_{21})}$ – in this case, the edge $k_{12}$ can be combined with any set of edges of ${\widetilde{G}}$ that do not contain a cycle;
- $\underline{\hbox { forests involving neither }\,k_{12}\,\hbox { nor }\,k_{21}}$ – these are spanning, incoming forests of $\widetilde{G}$.

Viewing each $c_i$ as such a sum exactly yields the formulas (15), as claimed.

Next, we note that

20 $$\begin{aligned} g_0~=~1 \quad \quad \textrm{and} \quad \quad g_{n-1}=0~, \end{aligned}$$

because (1) we have $\pi _F=1$ for the subgraph *F* with no edges, and (2) spanning, incoming forests of $\widetilde{G}$ have at most $n-2$ edges (and hence none have $n-1$ edges).

Next, it is straightforward to use the equations (15) and (20) to obtain the equation (17). To provide some detail, we use (15) to obtain expressions for $$k_{12}^{n-2} c_{n-1}$$, $$ k_{12}^{n-3} c_{n-2}$$, ..., $$ c_{1} $$, and then we observe that the terms involving $$g_i$$’s “telescope” as they are added together, so that subsequently what remains of these terms is $$k_{12}^{n-1}g_0 + g_{n-1} $$, which by (20), equals $$k_{12}^{n-1}$$.

We next prove the formula for the $g_i$’s given in (18). Recall that $g_i$ is the sum of all terms $\pi _F$, where *F* is a spanning incoming forest that is an *i*-edge subgraph of the star graph ${\widetilde{G}}$ in Figure 7. By definition, such forests contain at most 1 “outgoing” edge (i.e., an edge of the form $1 \rightarrow j$). Now it is straightforward to check that $e_{i}(\Sigma )$ is the subsum of $g_i$ corresponding to forests with no “outgoing” edges, and the remaining terms on the right-hand side of (18) form the subsum corresponding to forests with exactly 1 “outgoing” edge (a similar idea underlies the enumeration of spanning incoming forests shown in Figure 5).

Finally, the determinant of the matrix *M* is well known (see equation  (6) in the proof of (Gross et al. 2022, Theorem 6.1)). $\square$

We end this subsection by recalling a useful fact about elementary symmetric polynomials; this result is well known (cf. Gross et al. 2022, Proof of Theorem 6.3).

#### Lemma 3.8

Consider the following map, which evaluates a vector $(x_1,x_2,\dots ,x_n)$ at all elementary symmetric polynomials on the set $\{x_1,x_2,\dots ,x_n\}$:

$$\begin{aligned} {\mathbb {R}}^n ~&\rightarrow ~ {\mathbb {R}}^n \\ (x_1,x_2,\dots ,x_n) ~&\mapsto ~ \left( e_1(\{x_1,x_2,\dots ,x_n\}),~ e_2(\{x_1,x_2,\dots ,x_n\}), \right. \\&\quad \left. \dots , e_n(\{x_1,x_2,\dots ,x_n\})\right) ~. \end{aligned}$$

Then this map is generically *n*!-to-1.

### Identifiability of ${\mathcal {M}}_n(1,1)$

**Fig. 8:**
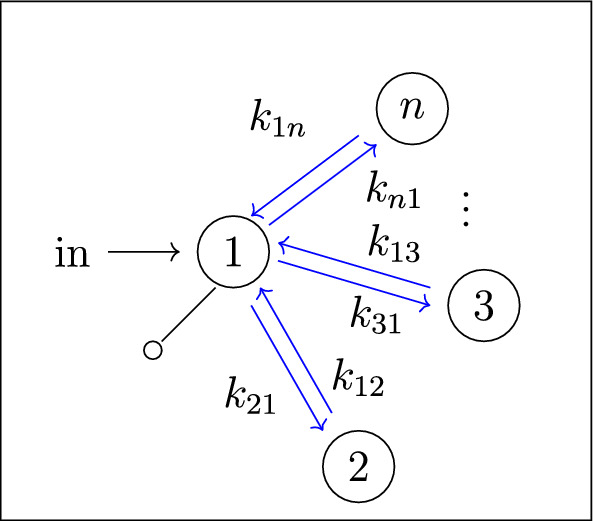
Parameter identifiability for ${\mathcal {M}}_n(1,1)$: all parameters are SLING

In this subsection, we show that all parameters are SLING in the mammillary model with input and output in the central compartment (see Figure 8).

#### Proposition 3.9

(${\mathcal {M}}_n(1,1)$) Let $n \ge 3$. If $\mathcal M =(G, \textit{In}, \textit{Out}, \textit{Leak})$ is an *n*-compartment mammillary model with no leaks ($\textit{Leak}= \varnothing$) and input and output in the central compartment ($\textit{In}=\textit{Out}=\{1\}$), then every parameter of ${\mathcal {M}}$ is SLING.

#### Proof

Every permutation of $\{2,3,\dots , n\}$ is a model automorphism of ${\mathcal {M}}$. Thus, the “incoming” edges $k_{12}, k_{13}, \dots , k_{1n}$ are symmetrically identical, as are the “outgoing” edges $k_{21}, k_{31}, \dots , k_{n1}$. Hence (and here $n \ge 3$ is used), Lemma 2.32 implies that every parameter is SLING or unidentifiable. However, Remark 3.1 implies that all parameters are at least locally identifiable. We conclude that all parameters are SLING. $\square$

#### Remark 3.10

Proposition 3.9 was proven previously (Cobelli et al. 1979) (using other methods), but we provide a proof here for completeness.

### Identifiability of ${\mathcal {M}}_n(1,2)$

**Fig. 9:**
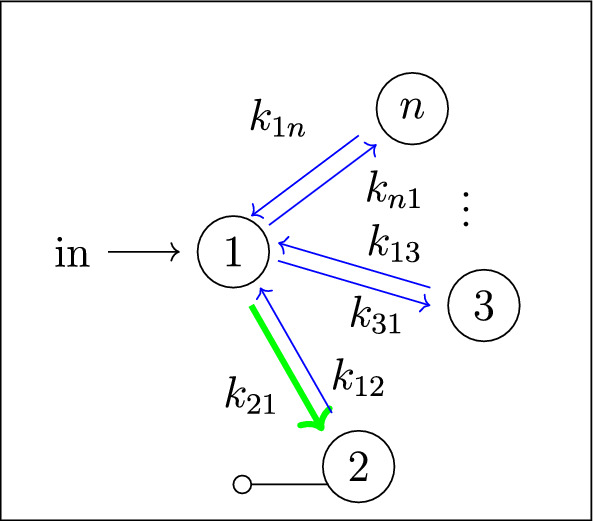
Parameter identifiability for ${\mathcal {M}}_n(1,2)$: the (green) boldface arrow indicates a globally identifiable parameter, and all others are SLING

For the 4-compartment model ${\mathcal {M}}_4(1,2)$, from Figure 2, we saw in Example 2.26 that the parameter $k_{21}$ is globally identifiable and all other parameters are SLING. This result generalizes to allow for more compartments, as follows (see Figure 9).

#### Proposition 3.11

(${\mathcal {M}}_n(1,2)$) Let $n \ge 4$. If $\mathcal M =(G, \textit{In}, \textit{Out}, \textit{Leak})$ is an *n*-compartment mammillary model with $\textit{In}=\{1\}$, $\textit{Out}=\{2\}$, and $\textit{Leak}= \varnothing$, then

1. the parameter $k_{21}$ is globally identifiable,
2. the parameter $k_{12}$ is SLING, and
3. the parameters $k_{13}, k_{14}, \dots , k_{1n}, ~ k_{31}, k_{41}, \dots , k_{n1}$ are SLING.

#### Proof

Part (1) follows directly from Lemma 2.27. Also, part (3) follows from Remark 3.1, Lemma 2.32, and the fact that every permutation of $\{3,4,\dots ,n\}$ is a model automorphism of ${\mathcal {M}}$ (here, the assumption $n \ge 4$ is used).

For part (2), we begin by analyzing the coefficients on the right-hand side of the input-output equation. By Proposition 2.11, these coefficients (denoted by $d_i$) are sums over certain subgraphs of the following graph $G_2^*$, which we obtain from the star graph in Figure 3 by removing the (unique) edge outgoing from compartment-2 (the output compartment): 
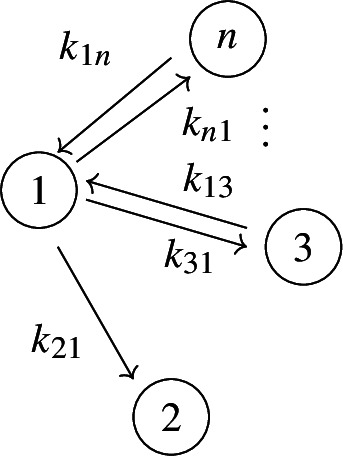
 Such subgraphs must connect the input and output compartments (in compartment-1 and compartment-2, respectively) and so must contain the edge $k_{21}$. Next, the condition of being an incoming forest precludes the remaining outgoing edges from compartment-1 (namely, $k_{31},k_{41},\dots , k_{n1}$). Thus, the coefficients $d_i$ are given by the following formulas involving elementary symmetric polynomials on the set $\Sigma :=\{k_{13}, k_{14}, \dots , k_{1n} \}$:

21 $$\begin{aligned} d_{n-2} ~&=~ k_{21}\nonumber \\ d_{n-3} ~&=~ k_{21}~ e_1(\Sigma ) \nonumber \\ d_{n-4} ~&=~ k_{21}~ e_2(\Sigma ) \nonumber \\&~ \vdots \nonumber \\ d_0 ~&=~ k_{21}~ e_{n-2}(\Sigma ) ~. \end{aligned}$$

Next, Proposition 3.7 gives the following equation, from (17), involving left-hand side coefficients $c_i$, the parameters $k_{12}$ and $k_{21}$, and elementary symmetric polynomials on $\Sigma$:

22 $$\begin{aligned}&k_{12}^{n-2} c_{n-1} - k_{12}^{n-3} c_{n-2} + k_{12}^{n-4} c_{n-3} - \dots \pm c_{1} \nonumber \\&\quad \quad ~=~ \left( k_{12}^{n-2} - k_{12}^{n-3}~ e_1(\Sigma ) + k_{12}^{n-4}~ e_2(\Sigma ) - \dots \pm e_{n-2}(\Sigma ) \right) k_{21} ~+~ k_{12}^{n-1}~. \end{aligned}$$

By moving all terms of equation (22) to the right-hand side, we obtain the following equation:

23 $$\begin{aligned} 0 ~&=~ k_{12}^{n-1} + ( k_{21} - c_{n-1}) k_{12}^{n-2} - ( k_{21} e_1(\Sigma ) -c_{n-2}) k_{12}^{n-3} + \dots \pm ( k_{21} e_{n-2}(\Sigma ) - c_{1}) \nonumber \\&=~ k_{12}^{n-1} + \sum _{i=2}^{n} (-1)^i ( k_{21} e_{i-2}(\Sigma ) -c_{n-i+1}) k_{12}^{n-i} ~, \end{aligned}$$

where we use the fact that $e_0(\Sigma ):=1$. Our aim is to apply Proposition 2.25 using the following function which is closely related to the right-hand side of (23):

24 $$\begin{aligned} h&~:=~ z^{n-1} + \sum _{i=2}^{n} (-1)^i ( k_{21} e_{i-2}(\Sigma ) -c_{n-i+1}) z^{n-i} ~. \end{aligned}$$

By construction (see equations (23)–(24)), $h|_{z=k_{12}} = 0$. Also, the leading coefficient (with respect to *z*) is 1, which is a nonzero function that is identifiable from the coefficient map (as in Definition 2.23). Therefore, to apply Proposition 2.25, we need only show that for all $i=2,3,\dots , n$, the polynomial function $(-1)^i ( k_{21} e_{i-2}(\Sigma ) - c_{n-i+1})$ is identifiable from the coefficient map. Indeed, the coefficients $c_i$ are (by definition) identifiable from the coefficient map, and we see from (21) that $k_{21}$ and the elementary symmetric polynomials $e_1(\Sigma ),e_2(\Sigma ),\dots , e_{n-2}(\Sigma )$ are identifiable from the coefficient map. Thus, we indeed can apply Proposition 2.25 and thereby conclude that $k_{12}$ is generically locally identifiable.

It remains only to show that $k_{12}$ is *not* generically globally identifiable. By Definition 2.18, it suffices to find a positive-measure subset $\Omega$ of the parameter space ${\mathbb {R}}^{|E|+ |\textit{Leak}|} = {\mathbb {R}}^{2n}$, such that, on $\Omega$, the relevant set arising from the coefficient map (namely, (9)) has size at least two.

As a step toward defining such a set $\Omega$, we revisit the function *h*, from (24). We rewrite *h* by using the equations (15) in straightforward manner to obtain the first equality here:

25 $$\begin{aligned} h&~=~ z^{n-1} + \sum _{i=2}^{n} (-1)^{i+1} ( k_{12} g_{i-2} + g_{i-1} ) z^{n-i} \nonumber ~\\&~=~ (z - k_{12}) ( z^{n-2} - g_1 z^{n-3} + g_2 z^{n-4}- \dots \pm g_{n-2} ) ~, \end{aligned}$$

and the second equality above uses the fact that $g_0=g_{n-1}=0$ (from Proposition 3.7).

Our next aim is to show that there is an open subset of parameter space ${\mathbb {R}}^{2n}$ on which, informally speaking, *h* always specializes to a (univariate) polynomial with $n-1$ distinct real roots. More precisely, we will show the existence of an open set $\Theta \subseteq {\mathbb {R}}^{2n}$ such that, for all $\textbf{k}^* \in \Theta$, the polynomial $h|_{\textbf{k}=\textbf{k}^*}$ (which is univariate in *z*) has $n-1$ distinct real roots.

We will construct such a set $\Theta$ as an open neighborhood of some vector $\mathbf {k^{\bullet }}$. To this end, we choose a vector of parameters $\mathbf {k^{\bullet }} = ( k^{\bullet }_{12}, k^{\bullet }_{21}, k^{\bullet }_{13}, k^{\bullet }_{31}, \dots , k^{\bullet }_{1n}, k^{\bullet }_{n1} ) \in {\mathbb {R}}^{2n}$ with two properties:

(i) $k^{\bullet }_{31}=k^{\bullet }_{41}=\dots =k^{\bullet }_{n1}=0$ (that is, all “outgoing” parameters, except $k_{21}$, are 0), and
(ii) the “incoming” parameters $k^{\bullet }_{12}, k^{\bullet }_{13}, k^{\bullet }_{14}, \dots , k^{\bullet }_{1n}$ are distinct.

We claim that $g_i|_{\textbf{k}=\mathbf {k^{\bullet }}} = e_i(\Sigma ^{\bullet })$ for all $i=1,2,\dots , n-2$, where $\Sigma ^{\bullet }:=\{k^{\bullet }_{13}, k^{\bullet }_{14}, \dots , k^{\bullet }_{1n} \}$. We verify this claim, as follows. First, recall from (16) that $g_i$ is a sum over incoming forests of the graph $\widetilde{G}$ in Figure 7. Thus, evaluating at $\textbf{k}^{\bullet }$ essentially disregards all “outgoing” edges in $\widetilde{G}$ (because the corresponding parameters are set to 0 by property (i)), leaving only “incoming” edges. These “incoming” edges correspond to the labels in $\Sigma ^{\bullet }$, which can be chosen freely to form incoming forests. We conclude that $g_i|_{\textbf{k}=\mathbf {k^{\bullet }}} = e_i(\Sigma ^{\bullet })$, as claimed.

Using (25), it now follows that:

26 $$\begin{aligned} h|_{\textbf{k}=\mathbf {k^{\bullet }}} ~=~ (z - k^{\bullet }_{12}) ( z^{n-2} - e_1(\Sigma ^{\bullet }) z^{n-3} + e_2(\Sigma ^{\bullet }) z^{n-4}- \dots \pm e_{n-2}(\Sigma ^{\bullet }) )~. \end{aligned}$$

It is easily seen from (26) that the set of roots of the polynomial $h|_{\textbf{k}=\mathbf {k^{\bullet }}}$ is $\{k^{\bullet }_{12}\} \cup \Sigma ^{\bullet }$ and therefore $h|_{\textbf{k}=\mathbf {k^{\bullet }}}$ has $n-1$ distinct real roots (property (ii) of $k^{\bullet }$ is used here). We claim that there is an open neighborhood $\Theta$ of $\mathbf {k^{\bullet }}$ such that for every $\mathbf {k^{*}} \in \Theta$, the polynomial $h|_{\textbf{k}={\mathbf {k^{*}}}}$ has $n-1$ distinct real roots. Indeed, this follows readily from two well-known facts: (1) the roots of a polynomial vary continuously in the coefficients of the polynomial (see, e.g., Rahman and Schmeisser 2002, Theorem 1.3.1, page 10), and (2) non-real roots of real-coefficient univariate polynomials come in complex-conjugate pairs.

Next, if needed, we shrink $\Theta$ to a smaller open neighborhood $\Omega$ of $\textbf{k}^{\bullet }$ so that property (ii) holds in the neighborhood. More precisely, we ensure that, for all $\mathbf {k^{*}}$ in the neighborhood $\Omega$, the parameter values $k^{*}_{12}, k^{*}_{13}, \dots , k^{*}_{1n}$ are distinct.

Now consider an arbitrary parameter vector $$\mathbf {k^{*}} $$ in $$\Omega $$. By Definition 2.18, it suffices to show the existence of another parameter vector $$\textbf{k}^{**} \in {\mathbb {R}}^{2n} $$, with $$k^{**}_{12} \ne k^*_{12}$$, such that both parameter vectors have the same image under the coefficient map, that is,

27 $$\begin{aligned} c_i(\textbf{k}^{*})~=~c_i(\textbf{k}^{**}) \quad \textrm{and} \quad d_j(\textbf{k}^{*})~=~d_j(\textbf{k}^{**}) \end{aligned}$$

for all $1 \le i \le n-1$ and $0 \le j \le n-2$, where the $c_i$’s and $d_j$’s are as in (15) and (21), respectively.

To this end, we begin to define such a vector $$\textbf{k}^{**}$$ by setting some of its coordinates equal to the corresponding values in $$\textbf{k}^{*}$$, as follows:

28 $$\begin{aligned} k^{**}_{21}~:=~k^*_{21} \quad \text {and} \quad (k^{**}_{13}, k^{**}_{14}, \dots , k^{**}_{1n}) ~:=~ ( k^{*}_{13},k^{*}_{14}, \dots , k^{*}_{1n})~. \end{aligned}$$

The above choice for $$k^{**}_{21}$$ is mandatory, as $$k_{21}$$ is globally identifiable. Also, the choices (28) guarantee that the parameters appearing in the formulas for $$d_0,d_1,\dots ,d_{n-2}$$, in (21), are the same in $$\textbf{k}^{*}$$ and $$\textbf{k}^{**}$$. Therefore, the desired equalities $$d_j(\textbf{k}^{*}) = d_j(\textbf{k}^{**})$$, from (27), hold for all $$0 \le j \le n-2$$.

The rest of this proof consists of defining the remaining coordinates of $$\textbf{k}^{**}$$ and then verifying the desired equalities $$c_i(\textbf{k}^{*})=c_i(\textbf{k}^{**}) $$ from (27).

To this end, consider the univariate polynomial $$h|_{\textbf{k}=\mathbf {k^{*}}}$$. One of its roots, by (25), is $$k^*_{12}$$. Additionally, there are $$n-2$$ additional (distinct) real roots (by construction of $$\Omega $$). Accordingly, we pick one of these roots and set $$k^{**}_{12}$$ equal to it. Hence, we have:

29 $$\begin{aligned} h|_{\textbf{k}=\mathbf {k^{*}}}(k^{**}_{12})=0 \quad \text {and} \quad k^{**}_{12} \ne k^*_{12}~. \end{aligned}$$

To define the remaining entries of $$\textbf{k}^{**}$$, we need to use a matrix that is obtained by specializing and performing row-operations on the matrix *M* in (19). To give details, consider the following $$(n-2) \times (n-2)$$ matrix:

30 $$\begin{aligned} M^* \quad := \quad M|_{k_{13}=k^*_{13},~k_{14}=k^*_{14},~\dots ,~ k_{1n}=k^*_{1n}}~. \end{aligned}$$

Next, let $\text {Shift}(M^*)$ be the $(n-2) \times (n-2)$ matrix obtained from $M^*$ by shifting the rows down by one; more precisely, the first row of $\text {Shift}(M^*)$ consists of 0’s, the second row is the first row of $M^*$, the third row is the second row of $M^*$, and so on, ending with row-$(n-2)$ being the row-$(n-3)$ of $M^*$. Finally, define:

31 $$\begin{aligned} \widetilde{M} \quad := \quad M^* + k^*_{12} \text {Shift}(M^*)~. \end{aligned}$$

We claim that $\widetilde{M}$ is invertible. To see this, recall from Proposition 3.7 that $\det M = \pm \prod _{3 \le j < \ell \le n} (k_{1j}- k_{1 \ell })$. Therefore, by (30), $\det M^* = \pm \prod _{3 \le j < \ell \le n} (k^*_{1j}- k^*_{1 \ell })$, which is nonzero (recall that the fact that $\mathbf {k^{*}}$ is in $\Omega$ guarantees that $k^{*}_{13}, k^{*}_{14}, \dots , k^{*}_{1n}$ are distinct). Hence, the matrix $M^*$ is invertible. Finally, by construction (31), $\widetilde{M}$ is obtained from $M^*$ by row operations and so is also invertible.

As $$\widetilde{M}$$ is invertible, we can now define the remaining entries of $$\textbf{k}^{**}$$, as follows, where we use the notation $$\Sigma ^*:=\{k^{*}_{13}, k^{*}_{14}, \dots , k^{*}_{1n}\}$$:32Next, we verify the equalities $$c_i(\textbf{k}^{*})=c_i(\textbf{k}^{**}) $$, in (27), for $$2 \le i \le n-1$$. Indeed, it is straightforward (although tedious) to check that these equalities follow readily from the formulas for $$c_2,c_3,\dots , c_{n-1}$$ in (15), the formulas for the $$g_i$$’s in terms of the matrix *M* in (18), the construction of the matrix $$\widetilde{M}$$ in (31), and our choice of parameters $$k_{ij}^{**}$$, including the formula (32).

Finally, it remains only to check that $$c_1(\textbf{k}^{*})=c_1(\textbf{k}^{**}) $$. To see this, we first recall from (29) that $$h|_{\textbf{k}=\mathbf {k^{*}}}(k^{**}_{12})=0$$. Thus, by using (24) (and comparing with (23)), we obtain the following:

33 $$\begin{aligned} 0 ~&=~ (k^{**}_{12})^{n-1} + ( k^{*}_{21} - c^{*}_{n-1}(\textbf{k}^{*})) (k^{**}_{12})^{n-2} - ( k^{*}_{21} e_1(\Sigma ^*) -c^{*}_{n-2}(\textbf{k}^{*})) (k^{**}_{12})^{n-3} + \dots \nonumber \\ ~&\quad \pm ( k^{*}_{21} e_{n-2}(\Sigma ^*) - c^{*}_{1}(\textbf{k}^{*})) ~. \end{aligned}$$

Next, consider specializing the equation (23) at $$\textbf{k}=\mathbf {k^{**}}$$. We claim that the resulting equation is exactly what is shown in  (33), except that $$c_1(\textbf{k}^{*}) $$ (in the second line) is replaced by $$c_1(\textbf{k}^{**})$$. Indeed, this claim follows readily from two facts: (1) the parameter $$k_{21}$$ and the parameters in $$\Sigma $$ are the same between $$\mathbf {k^{*}}$$ and $$\mathbf {k^{**}}$$ (recall (28)), and (2) $$c_i(\textbf{k}^{*})=c_i(\textbf{k}^{**}) $$, for $$2 \le i \le n-1$$, which we showed above. We therefore conclude that $$c_1(\textbf{k}^{*})=c_1(\textbf{k}^{**}) $$. $$\square $$

### Identifiability of ${\mathcal {M}}_n(2,1)$

**Fig. 10:**
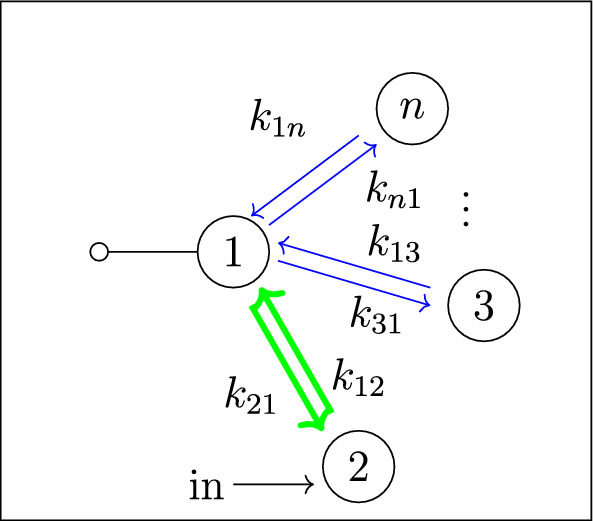
Parameter identifiability for ${\mathcal {M}}_n(2,1)$: the (green) boldface arrows indicate generically globally identifiable parameters (in fact, $k_{12}$ is globally identifiable), and all others are SLING

In this subsection, we analyze mammillary models with input in a peripheral compartment and output in the central compartment (Figure 10). The proof of Proposition 3.12 below is similar to certain parts of the proof we gave above for Proposition 3.11.

#### Proposition 3.12

(${\mathcal {M}}_n(2,1)$) Let $n \ge 4$. If $\mathcal M =(G, \textit{In}, \textit{Out}, \textit{Leak})$ is an *n*-compartment mammillary model with $\textit{In}=\{2\}$, $\textit{Out}=\{1\}$, and $\textit{Leak}= \varnothing$, then

1. the parameter $k_{12}$ is globally identifiable,
2. the parameter $k_{21}$ is generically globally identifiable, and
3. the parameters $k_{13}, k_{14}, \dots , k_{1n}, ~ k_{31}, k_{41}, \dots , k_{n1}$ are SLING.

#### Proof

Part (1) follows directly from Lemma 2.27 ($k_{12}$ equals the coefficient $d_{n-2}$). Also, part (3) follows from Remark 3.1, Lemma 2.32, and the fact that every permutation of $\{3,4,\dots ,n\}$ is a model automorphism of ${\mathcal {M}}$ (here, the assumption $n \ge 4$ is used).

For part (2), we begin by analyzing the coefficients on the right-hand side of the input-output equation. According to Proposition 2.11, these coefficients are sums over certain subgraphs of the following graph $G_1^*$, which is obtained from the star graph in Figure 3 by removing all the edges outgoing from compartment-1 (the output compartment):
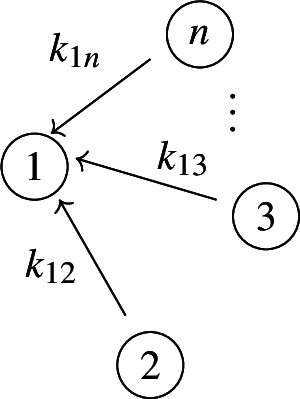
 In order to connect the input and output compartments (in compartment-2 and compartment-1, respectively), the subgraphs must contain the edge $k_{12}$. The condition of being an incoming forest is vacuous (in the graph above, no compartment has more than one outgoing edge, nor can any of the edges form a cycle). We conclude that $k_{12}$ is a factor of every right-hand side coefficient $d_i$, as in (8), and moreover these coefficients $d_i$ are given by the following formulas involving elementary symmetric polynomials on the set $\Sigma :=\{k_{13}, k_{14}, \dots , k_{1n} \}$:

34 $$\begin{aligned} d_{n-2} ~&=~ k_{12}\nonumber \\ d_{n-3} ~&=~ k_{12}~ e_1(\Sigma ) \nonumber \\ d_{n-4} ~&=~ k_{12}~ e_2(\Sigma ) \nonumber \\&~ \vdots \nonumber \\ d_0 ~&=~ k_{12}~ e_{n-2}(\Sigma ) ~=~ k_{12} k_{13} \dots k_{1n}~. \end{aligned}$$

Next, Proposition 3.7 yields an equation (17) involving left-hand side coefficients $c_i$, which we reproduce here for convenience:

35 $$\begin{aligned}&k_{12}^{n-2} c_{n-1} - k_{12}^{n-3} c_{n-2} + k_{12}^{n-4} c_{n-3} - \dots \pm c_{1} \nonumber \\&\quad \quad ~=~ \left( k_{12}^{n-2} - k_{12}^{n-3}~ e_1(\Sigma ) + k_{12}^{n-4}~ e_2(\Sigma ) - \dots \pm e_{n-2}(\Sigma ) \right) k_{21} ~+~ k_{12}^{n-1}~. \end{aligned}$$

The idea now is to view equation (35) as a linear polynomial in $k_{21}$ with coefficients that are identifiable from the coefficient map. More precisely, we use the equations (34) to perform the following steps to obtain a nonzero linear polynomial in $k_{21}$ whose coefficients are polynomial expressions in the $c_i$’s and $d_i$’s (and thus are identifiable from the coefficient map):

1. Multiply both sides by $k_{12}$, and then replace $k_{12}^{n-2}~ e_1(\Sigma )$ by $k_{12}^{n-3}~d_{n-3}$, and so on, ending by replacing $k_{12}~ e_{n-2}(\Sigma )$ by $d_0$;
2. Replace each occurrence of $k_{12}$ by $d_{n-2}$.

Now Proposition 2.25 applies, and so $k_{21}$ is generically globally identifiable. $\square$

### Identifiability of ${\mathcal {M}}_n(2,2)$

**Fig. 11:**
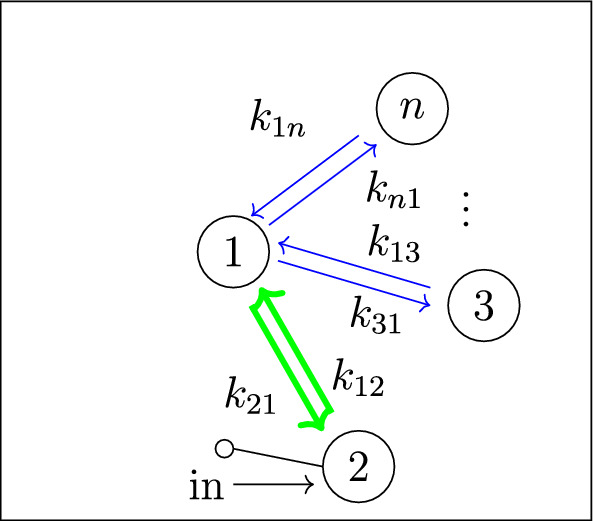
Parameter identifiability for ${\mathcal {M}}_n(2,2)$: the (green) boldface arrows indicate generically globally identifiable parameters (in fact, $k_{12}$ is globally identifiable), and all others are SLING

This subsection focuses on mammillary models in which the input and output are in the same peripheral compartment. We show that the edges incident to this peripheral compartment have generically globally identifiable parameters, and all other parameters are SLING (see Figure 11). We motivate this result – and the idea behind the proof – through the following example.

#### Example 3.13

By Proposition 2.11, the coefficients of the input-output equation for the model ${\mathcal {M}}_4(2,2)$ are as follows:

$$\begin{aligned} c_3 ~&=~k_{12} + k_{13} + k_{14} + k_{21} + k_{31} + k_{41} \\ c_2 ~&=~k_{12}k_{13}+k_{12}k_{14}+ k_{12}k_{31}+k_{12}k_{41}+k_{13}k_{14}+k_{13}k_{21} \\&\quad +k_{13}k_{41} +k_{14}k_{21}+k_{14}k_{31} \\ c_1 ~&=~k_{12}k_{13}k_{14}+k_{12}k_{13}k_{41}+k_{12}k_{14}k_{31}+k_{13}k_{14}k_{21} \\ d_2 ~&=~k_{13}+k_{14}+k_{21}+k_{31}+k_{41} \\ d_1 ~&=~k_{13}k_{14}+k_{13}k_{21}+k_{13}k_{41}+k_{14}k_{21}+k_{14}k_{31} \\ d_0 ~&=~ k_{13}k_{14} k_{21} \end{aligned}$$

It is straightforward to check that the difference between the two linear coefficients, $c_3-d_2$, equals $k_{12}$ and so this parameter is globally identifiable. Additionally, the difference between the degree-two coefficients, $c_2-d_1$, is a multiple of $k_{12}$ and, indeed, equals $k_{12}(d_2-k_{21})$. As we already saw that $k_{12}$ is globally identifiable, we conclude that $k_{21}$ is generically globally identifiable (“generically” is here because, when $k_{12}=0$, it may not be possible to recover $k_{21}$). These ideas generalize, as seen in the next result (equation (36) in particular).

#### Proposition 3.14

(${\mathcal {M}}_n(2,2)$) Let $n \ge 4$. If $\mathcal M =(G, \textit{In}, \textit{Out}, \textit{Leak})$ is an *n*-compartment mammillary model with $\textit{In}=\{2\}$, $\textit{Out}=\{2\}$, and $\textit{Leak}= \varnothing$, then

1. the parameters $k_{12}$ and $k_{21}$ are generically globally identifiable (in fact, $k_{12}$ is globally identifiable), and, moreover, their values can be determined by the following formulas:
  36 $$\begin{aligned} k_{12} ~&=~ c_{n-1}-d_{n-2}~, \quad \textrm{and} \nonumber \\ k_{21} ~&=~ d_{n-2} - \frac{c_{n-2}-d_{n-3}}{k_{12}} ~=~ d_{n-2} - \frac{c_{n-2}-d_{n-3}}{c_{n-1}-d_{n-2}} ~, \end{aligned}$$
  where $c_i$ and $d_i$ are the coefficients of the input-output equation, as in (7)–(8); and
2. the parameters $k_{13}, k_{14}, \dots , k_{1n}, ~ k_{31}, k_{41}, \dots , k_{n1}$ are SLING.

#### Proof

Part (2) follows from Remark 3.1, Lemma 2.32, and the fact that every permutation of $\{3,4,\dots ,n\}$ is a model automorphism of ${\mathcal {M}}$ (here, the assumption $n \ge 4$ is used).

For part (1), we will use the formula for coefficents of the input-output equation (Proposition 2.11). In our model ${\mathcal {M}} = (G, \textit{In}, \textit{Out}, \textit{Leak})$, the input and output are in the same compartment, so it is vacuously true that the input and output are always connected in spanning subgraphs of $G^*_2$ (recall that $G^*_2$ is obtained from *G* by removing the outgoing edge $k_{12}$ from the output compartment-2). Therefore, the subgraph-connectedness condition for the right-hand side coefficients $d_i$, in (8), can be safely ignored in what follows.

We first analyze $k_{12}$. By Proposition 2.11, the linear-polynomial coefficient on the left-hand side, namely, $c_{n-1}$, is a sum over all edges of *G*. On the other hand, the corresponding coefficient on the right-hand side, $d_{n-2}$, is a sum over all edges except $k_{12}$ (because it was removed from *G* to obtain $G^*_2$). We conclude that $k_{12} = c_{n-1}-d_{n-2}$, as desired.

Now we consider $k_{21}$. This time we focus on the degree-two coefficients in Proposition 2.11, namely, $c_{n-2}$ and $d_{n-3}$. Their difference is a sum over all 2-edge spanning incoming forests of the mammillary graph such that one of the edges is $k_{12}$. The remaining edge in such a subgraph is any edge besides $k_{12}$ and $k_{21}$. We therefore obtain the first equality here:

37 $$\begin{aligned} c_{n-2} - d_{n-3} ~&=~ k_{12} (k_{13}+k_{14}+ \dots + k_{1n} ~+~ k_{31}+k_{41}+ \dots + k_{n1}) \nonumber \\ ~&=~ k_{12} (d_{n-2}-k_{21})~, \end{aligned}$$

and the second equality above follows from the description of $k_{21}$ given earlier in the proof.

Now it is straightforward to solve for $k_{21}$ in (37) and obtain the desired formula (36). $\square$

### Identifiability of ${\mathcal {M}}_n(2,3)$

**Fig. 12:**
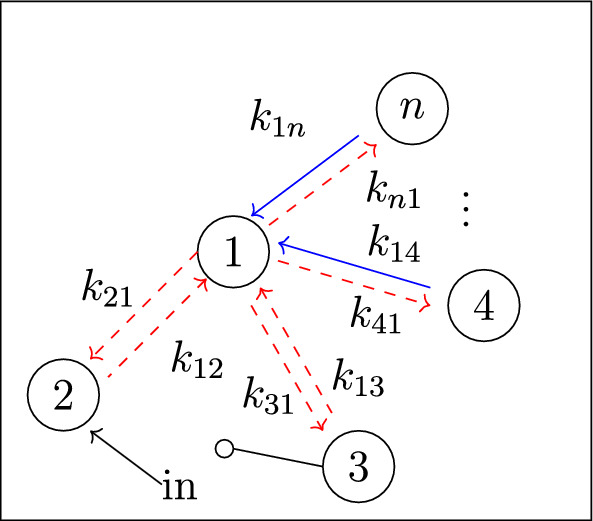
Parameter identifiability for ${\mathcal {M}}_n(2,3)$: the red dashed arrows indicate parameters that we conjecture to be unidentifiable, and all others are SLING

As noted earlier in Remark 3.1, the model ${\mathcal {M}}_n(2,3)$ contains unidentifiable parameters. Some parameters, however, are SLING; these parameters correspond to certain edges directed toward to the central compartment (see the [blue] solid arrows in Figure 12). To illustrate the ideas behind the proof of this result (Proposition 3.16 below), we present the following example.

#### Example 3.15

For the model ${\mathcal {M}}_5(2,3)$, it is straightforward to apply Proposition 2.11 to obtain the following coefficients on the right-hand side of the input-output equation:

38 $$\begin{aligned} d_3 ~&=~0 \nonumber \\ d_2 ~&=~ k_{12}k_{31} \nonumber \\ d_1 ~&=~ k_{12}k_{31} (k_{14}+k_{15}) \nonumber \\ d_0 ~&=~ k_{12}k_{31} (k_{14} k_{15})~. \end{aligned}$$

We see from the coefficients (38) that the following elementary symmetric polynomials are identifiable from the coefficient map: $e_1(\Sigma ')= k_{14}+k_{15}$ and $e_2(\Sigma ') = k_{14}k_{15}$, where $\Sigma ' = \{k_{14}, k_{15} \}$. This allows us to recover the set $\Sigma '$, but the symmetry between compartment-4 and compartment-5 precludes us from distinguishing between $k_{14}$ and $k_{15}$. We conclude that the parameters $k_{14}$ and $k_{15}$ are SLING.

#### Proposition 3.16

(${\mathcal {M}}_n(2,3)$) Let $n \ge 5$. If $\mathcal M =(G, \textit{In}, \textit{Out}, \textit{Leak})$ is an *n*-compartment mammillary model with $\textit{In}=\{2\}$, $\textit{Out}=\{3\}$, and $\textit{Leak}= \varnothing$, then the parameters $k_{14}, k_{15}, \dots , k_{1n}$ are SLING.

#### Proof

Every permutation of $\{4,5,\dots ,n\}$ is a model automorphism of ${\mathcal {M}}$. Hence, by Proposition 2.32, the parameters $k_{14}, k_{15}, \dots , k_{1n}$ are either all SLING or all unidentifiable (here, the assumption $n \ge 5$ is used). Therefore, it suffices to show that $k_{14}, k_{15}, \dots , k_{1n}$ are generically locally identifiable.

Let $\Sigma ':=\{k_{14}, k_{15}, \dots , k_{1n} \}$. We claim that the right-hand side coefficients, $d_i$, as in (8), can be written in terms of elementary symmetric polynomials on $\Sigma '$, as follows:

39 $$\begin{aligned} d_{n-2} ~&=~ 0 \nonumber \\ d_{n-3} ~&=~ k_{12}k_{31} \nonumber \\ d_{n-4} ~&=~ k_{12}k_{31}~e_1(\Sigma ') \nonumber \\ d_{n-5} ~&=~ k_{12}k_{31}~e_2(\Sigma ') \nonumber \\ ~&~\vdots \nonumber \\ d_{0} ~&=~ k_{12}k_{31}~e_{n-3}(\Sigma ') ~. \end{aligned}$$

(These equations (39) in the case of $n=5$ were shown earlier in (38).)

We verify the formulas (39) by using Proposition 2.11, as follows. First, the input and output are in distinct peripheral compartments, so they can not be connected by a single edge. Thus, $d_{n-2} = 0$. Next, the only spanning incoming forests with two edges that connect the input to the output here are $k_{12}k_{31}$ and $k_{21}k_{13}$, and since the edge $k_{13}$ is deleted in $G^*$, we must have $d_{n-3}=k_{12}k_{31}$. Similarly, all terms in the remaining coefficients $d_i$, for $i=n-2,n-1,\dots , 0$, must have a factor of $k_{12}k_{31}$, since this is the only pair of edges that can take part in a spanning incoming forest that connects the input and output. If we begin with $k_{12}$ and $k_{31}$ and then try to add more edges, the only edges we can add without creating cycles or having more than one outgoing edge from compartment-1 are the edges of $\Sigma ' = \{k_{14}, k_{15}, \dots , k_{1n}\}$. Indeed, such edges can be added arbitrarily, which yields the elementary symmetric polynomials shown in (39).

Next, the equations (39) imply that the elementary symmetric polynomials appearing in these equations, namely, $e_1(\Sigma '), e_2(\Sigma '), \dots , e_{n-3}(\Sigma ')$, are identifiable from the coefficient map. Now Lemma 3.8 implies that each parameter $k_{14}, k_{15}, \dots , k_{1n}$, when viewed as a (projection) function ${\mathbb {R}}^{|E| + |\textit{Leak}|} \rightarrow {\mathbb {R}}$, is locally identifiable from the coefficient map. Hence, by Remark 2.24, each of the parameters $k_{14}, k_{15}, \dots , k_{1n}$ is generically locally identifiable. $\square$

#### Remark 3.17

(${\mathcal {M}}_4(2,3)$) The ideas in the proof of Proposition 3.16 can be used to show that, in the $n=4$ version of the model ${\mathcal {M}}_n(2,3)$, the parameter $k_{14}$ is generically globally identifiable. Indeed, in this case, the equations (39) imply that $k_{14}= \frac{d_0}{d_1}$.

Next, we conjecture that all parameters not considered in Proposition 3.16 are unidentifiable, as follows (cf. Figure 12).

#### Conjecture 3.18

(${\mathcal {M}}_n(2,3)$) Let $n \ge 5$. If $\mathcal M =(G, \textit{In}, \textit{Out}, \textit{Leak})$ is an *n*-compartment mammillary model with $\textit{In}=\{2\}$, $\textit{Out}=\{3\}$, and $\textit{Leak}= \varnothing$, then the following parameters are unidentifiable:

$$\begin{aligned} k_{12}~, \quad k_{21}~, \quad k_{13}~, \quad \textrm{and} \quad k_{31}~, k_{41}, \dots , k_{n1}~. \end{aligned}$$

#### Remark 3.19

By Lemma 2.32, the parameters $k_{41}, k_{51}, \dots , k_{n1}$ in the model ${\mathcal {M}}_n(2,3)$ all have the same type of identifiability. Also, the equation $d_{n-3}=k_{12} k_{31}$, from equation (39) in the proof of Proposition 3.16, implies that $k_{12}$ and $k_{31}$ have the same type of identifiability. Conjecture 3.18 asserts that all of these parameters are, in fact, unidentifiable.

## Discussion

In this work, we prove the identifiability properties of individual parameters (specifically, generically globally identifiable versus SLING) in mammillary models with one input, one output, and no leaks (Theorem 3.2). As noted earlier, we are essentially the first to undertake such an investigation for an infinite family of models. We therefore expect that our work to lead the way for future studies, involving other families of models.

A natural candidate for such future investigations is the family of *catenary models*, those in which the underlying graph is a bidirected path (see (Cobelli et al. 1979) and (Cobelli et al. 2002, §5.8) for partial results in this direction). A starting point for such a project is the database of catenary models with three compartments that appears in the recent work of Dessauer *et al.* (2024, Appendix B). In this database, each model is shown together with the identifiability properties of all parameters. We also direct the reader to additional databases of linear compartmental models, one for models with three compartments (created by Norton) (Norton 1982) and one for models with up to four compartments (created by Gogishvili) (Gogishvili 2024).

Returning our attention to mammillary models, there are a number of future directions. The first is to resolve Conjecture 3.18, which pertains to the parameters in model ${\mathcal {M}}_n(2,3)$ that we believe are unidentifiable (as shown in Figure 1). Additionally, it is natural to ask how our results extend to allow for (mammillary models with) any number of inputs, outputs, and leaks. In other words, how does Theorem 3.2 generalize when inputs, output, and/or leaks are added? This question is open, although some partial results were proven by Chau (1985b) and Cobelli et al. (1979) (see also (Cobelli et al. 2002, §5.8)).

A version of this question – at the level of models rather than their parameters – has been addressed in general (Chan et al. 2021; Gerberding et al. 2020; Gogishvili 2024; Gross et al. 2019), but we are not aware of results in this direction pertaining to individual parameters. The aforementioned questions – which investigate the effect on identifiability of various operations on models – are important in the context of systems biology, because a model might not be completely known or we may want to predict the effect of various interventions that lead to changes in the model. For instance, we might not know precisely which compartments have leaks, and so we would want to answer the question of identifiability for a family of models arising from various possible sets of leaks. Additionally, although we considered models without leaks in this work, we recognize that models that do contain leaks are more realistic in biological settings, as there are very few systems that are completely closed. Finally, allowing for more inputs or outputs represents additional controls or measurements of the system, which again may better reflect the situation in experimental settings. In short, our results take the first step into an investigation with direct relevance for biological applications.

## Data Availability

This work does not have any associated data.
